# Cross‐Strand Chimeric RNA Signature Predicts Prognosis and Identifies Tumor Immune Microenvironment Associations in Gastric Cancer

**DOI:** 10.1155/humu/4428673

**Published:** 2026-06-17

**Authors:** Shuqiang Cheng, Miaomiao Cui, Xuehui Li, Yun Huang, Xianhui Wen, Sixi Wei, Shaocun Zhang, Hai Huang

**Affiliations:** ^1^ Center for Clinical Laboratories, The Affiliated Hospital of Guizhou Medical University, Guiyang, China, gmcah.cn; ^2^ School of Clinical Laboratory Science, Guizhou Medical University, Guiyang, China, gmc.edu.cn; ^3^ Department of Clinical Laboratory, The Affiliated Jinyang Hospital of Guizhou Medical University, Guiyang, China; ^4^ Department of Rheumatology and Immunology, Peking University Third Hospital, Beijing, China, puh3.net.cn

**Keywords:** CIBERSORT, cross-strand chimeric RNA, gastric cancer, immunosuppression, machine learning, prognostic biomarker, somatic mutation, translational regulation, tumor immune microenvironment, tumor mutation burden

## Abstract

Cross‐strand chimeric RNAs (cscRNAs) represent an emerging class of noncanonical fusion transcripts arising from bidirectional transcription, yet their landscape and functional significance in gastric cancer remain unexplored. Here, we systematically investigated cscRNA expression patterns, prognostic relevance, and tumor immune microenvironment associations in gastric cancer through integrated computational and experimental approaches. Analysis of 372 TCGA‐STAD samples revealed tumor‐specific cscRNA enrichment that was independent of clinicopathological variables including tumor stage, histological grade, and lymph node metastasis status. Through a three‐layer machine learning pipeline integrating univariate screening, multivariate feature selection, and bootstrap stability validation, we identified six cscRNA‐enriched genomic regions that collectively formed a reproducible prognostic signature. Patients stratified by the risk score model showed significantly divergent survival outcomes (median survival: 18.9 vs. 69.0 months; univariate HR = 2.54, *p* < 0.001), and the risk score retained independent prognostic value after adjusting for clinical covariates (multivariable HR = 2.86, *p* < 0.001). In an exploratory analysis of an independent validation cohort (GSE122401, *n* = 26), high‐risk tumors showed features consistent with an immunosuppressive microenvironment, including reduced CD8+ T‐cell infiltration (*p* = 0.042), elevated regulatory T‐cell proportions (*p* = 0.028), and lower immune scores (*p* = 0.006). Subgroup analyses by microsatellite instability and Epstein–Barr virus status (*n* = 4 and *n* = 6, respectively) suggested differential immune patterns warranting confirmation in larger cohorts. Experimental validation confirmed cscR‐819 as a bona fide posttranscriptional cscRNA product, whose knockdown significantly inhibited gastric cancer cell proliferation, migration, and colony formation while promoting apoptosis. Mechanistically, cscR‐819 was predominantly localized in the cytoplasm and functioned as a translational regulator, selectively enhancing the translation efficiency of genes involved in cell cycle progression, DNA replication, and antiapoptotic pathways, as demonstrated by polyribosome profiling. In conclusion, our study establishes cscRNAs as functionally relevant contributors to gastric cancer pathogenesis and identifies exploratory associations with tumor immune microenvironment features, positioning cscRNA‐based signatures as promising candidate biomarkers for risk stratification and motivating further investigation of cscRNA–immune interactions.

## 1. Introduction

Gastric cancer (GC) remains the fifth most commonly diagnosed malignancy and the fourth leading cause of cancer‐related mortality worldwide, with over 1 million new cases and approximately 769,000 deaths reported annually [1]. Despite advances in multimodal treatment strategies, the 5‐year survival rate for advanced GC remains below 30%, largely due to late‐stage diagnosis, high metastatic potential, and marked clinical heterogeneity [2]. Current prognostic stratification relies primarily on the American Joint Committee on Cancer (AJCC) TNM staging system, which assesses tumor anatomical extent, lymph node involvement, and distant metastasis [3]. However, patients within the same pathological stage often exhibit vastly different clinical outcomes, underscoring the inadequacy of anatomical classification alone and highlighting the urgent need for molecular biomarkers that capture tumor biological behavior [4]. The transcriptome, as the functional intermediary between genome and proteome, exhibits remarkable complexity beyond protein‐coding genes, encompassing diverse RNA species generated through alternative splicing, RNA editing, and bidirectional transcription [5]. Emerging evidence suggests that noncanonical transcriptional events—including antisense transcription, intergenic transcription, and cross‐strand chimeric RNA (cscRNA) formation—may play previously underappreciated roles in cancer pathogenesis [6]. Understanding these unconventional transcriptomic features may reveal novel regulatory mechanisms underlying GC progression and provide new avenues for precision oncology.

cscRNAs represent an emerging class of noncanonical fusion transcripts arising from bidirectional transcription on opposite DNA strands within the same genomic locus [7]. Unlike conventional intergenic fusion RNAs generated by chromosomal rearrangements or trans‐splicing events, cscRNAs originate from convergent transcription initiated from both forward and reverse strands, resulting in chimeric molecules that contain sequences from opposing genomic orientations [8]. Through systematic analysis of paired‐end RNA sequencing data, Peng and colleagues first demonstrated the widespread existence of cscRNAs in human cells, identifying these fusion events as products of cotranscriptional RNA–RNA ligation or posttranscriptional splicing between bidirectionally transcribed transcripts [7]. This bidirectional transcriptional architecture is facilitated by head‐to‐head gene arrangements, divergent promoters, or cryptic antisense promoters within gene bodies, which are increasingly recognized as pervasive features of mammalian genomes [9, 10]. Subsequent studies have detected cscRNAs across various tissue types and cellular contexts, suggesting context‐dependent expression patterns and potential regulatory functions [11]. In cancer contexts, aberrant bidirectional transcription has been linked to oncogenic activation, with dysregulated sense–antisense transcript pairs implicated in hepatocellular carcinoma, lung cancer, and colorectal cancer progression [12, 13]. However, despite these foundational observations, the landscape of cscRNA expression in GC remains entirely uncharacterized. Critical questions persist: Do cscRNAs exhibit tumor‐specific expression patterns in GC? Are they associated with clinicopathological features or patient prognosis? Most fundamentally, what are the molecular mechanisms through which individual cscRNAs contribute to GC pathogenesis? Addressing these gaps requires systematic profiling of cscRNA repertoires in large patient cohorts, identification of functionally relevant candidates, and mechanistic dissection of their roles in tumor biology.

To address these critical knowledge gaps, we conducted a comprehensive investigation of cscRNAs in GC through integrated computational and experimental approaches. Using RNA sequencing data from 372 gastric adenocarcinoma samples in The Cancer Genome Atlas (TCGA‐STAD) cohort, we systematically profiled cscRNA expression patterns and evaluated their associations with clinicopathological variables and patient outcomes. To overcome the inherent instability of individual cscRNA detection, we implemented a genomic region–based aggregation strategy, defining 5‐kb bins across the genome to quantify cumulative cscRNA activity and identify recurrent hotspots. Through a three‐layer machine learning pipeline integrating univariate statistical screening, multivariate feature selection, and bootstrap stability validation, we identified six reproducible cscRNA‐enriched genomic regions associated with patient prognosis. Critically, we constructed a multiregion risk score model that demonstrated independent prognostic value in multivariable analysis. To further explore the biological implications of cscRNA‐based risk stratification, we characterized its association with the tumor immune microenvironment using an independent Gene Expression Omnibus (GEO) validation cohort, applying CIBERSORT immune cell deconvolution and Estimation of STromal and Immune cells in MAlignant Tumor tissues using Expression data (ESTIMATE) algorithms to characterize immune microenvironment features associated with risk groups. To validate computational predictions and elucidate functional mechanisms, we performed RT‐PCR validation of candidate cscRNAs in GC cell lines, followed by comprehensive functional assays and polyribosome profiling to dissect molecular mechanisms. Our findings reveal that cscRNAs represent a previously unrecognized layer of transcriptomic complexity in GC, with specific cscRNA molecules functioning as translational regulators that selectively enhance expression of proliferation‐ and survival‐associated genes. Moreover, in an exploratory analysis of an independent validation cohort, cscRNA‐based risk stratification was associated with tumor immune microenvironment features compatible with an immunosuppressive phenotype, including reduced cytotoxic T‐cell infiltration and elevated regulatory T‐cell presence (interpreted as exploratory and warranting confirmation in larger cohorts). This work not only expands our understanding of noncanonical transcription in GC pathogenesis but also establishes cscRNA‐based signatures as promising biomarkers for risk stratification, provides mechanistic insights into posttranscriptional regulation, and identifies exploratory associations between cscRNA dysregulation and tumor immune microenvironment features that motivate further investigation of cscRNA–immune interactions.

## 2. Methods

### 2.1. Data Source and Sample Information

We obtained gastric adenocarcinoma (STAD) RNA sequencing data and corresponding clinical information from The Cancer Genome Atlas (TCGA) database. The dataset comprised 372 samples, including 331 primary tumor samples and 41 solid tissue normal samples. For survival analysis, we analyzed 307 GC patients from the TCGA‐STAD cohort with complete survival data, including overall survival time (months) and vital status (alive/deceased). Clinical variables analyzed included gender (male/female), age at diagnosis (categorized as < 50, 50–64, 65–74, and ≥ 75 years), AJCC tumor stage (Stages I–IV), histological grade (G1–G3), and lymph node metastasis status (N0 vs. N+).

### 2.2. cscRNA Detection and Quantification

cscRNAs were identified from RNA‐seq data as previously described [7]. Briefly, cscRNAs represent fusion transcripts arising from bidirectional transcription on opposite DNA strands. The number of cscRNAs detected in each sample was quantified and used as the primary metric for analysis. For genomic region analysis, expression levels of 103 cscRNA‐enriched genomic regions were quantified as reads per million (RPM) and log_10_‐transformed (log_10_(RPM + 1)) to handle skewed distributions and zero values.

### 2.3. Statistical Analysis of cscRNA Patterns

**Figure 1:**
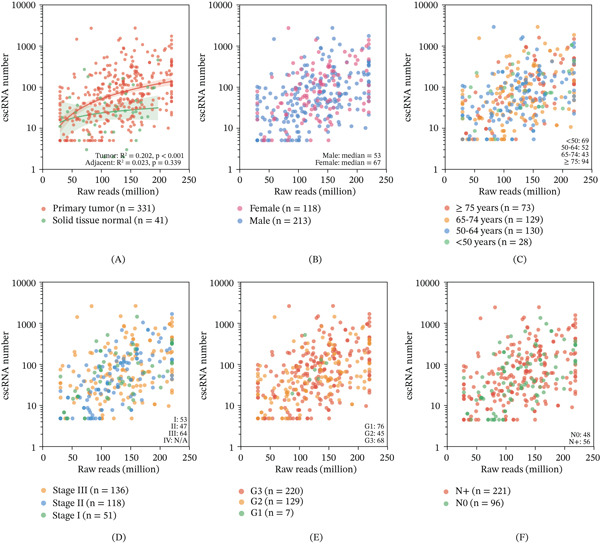
Tissue‐specific cscRNA detection patterns and independence from clinicopathological features in gastric cancer. (A) Relationship between sequencing depth and cscRNA abundance in gastric cancer samples from the TCGA‐STAD cohort. A scatter plot displays individual samples with the *x*‐axis representing raw sequencing reads (millions) and the *y*‐axis representing cscRNA number on a log_10_ scale. Primary tumor tissues are shown in red; solid tissue normal samples are shown in green. Solid lines represent linear regression fits; shaded areas indicate 95% confidence intervals. Pearson correlation coefficients (*R* ^2^) and *p* values are displayed for each tissue type. (B–F) cscRNA distribution in tumor samples stratified by clinicopathological features. In all panels, axes and median annotations follow Figure 1A; group‐comparison *p* values are displayed within each plot. (B, F) Two‐group comparisons used a two‐tailed Mann–Whitney *U* test; (C–E) multigroup comparisons used the Kruskal–Wallis test. (B) Patient gender (male, blue; female, pink).

To assess the relationship between cscRNA abundance and sequencing depth, we performed linear regression analysis separately for tumor and normal tissue samples, calculating Pearson correlation coefficients (*R* ^2^) and associated *p* values. To evaluate associations between cscRNA levels and clinicopathological features, we employed nonparametric statistical tests: Mann–Whitney *U* test for two‐group comparisons (gender and lymph node status) and Kruskal–Wallis *H* test for multigroup comparisons (age groups, tumor stage, and histological grade). Statistical significance was defined as *p* < 0.05. Because cscRNA counts in tumor tissues correlated positively with sequencing depth (Figure 1A), we explicitly examined whether sequencing depth differed across the clinical subgroups before drawing inferences about cscRNA abundance. Raw sequencing read counts were compared across gender, age, AJCC stage, histological grade, and lymph node status using the same Mann–Whitney *U* or Kruskal–Wallis tests applied to cscRNA counts (all *p* > 0.10), confirming that sequencing depth was not systematically biased between subgroups. As an additional sensitivity analysis, partial Spearman correlations between cscRNA counts and each ordinal clinical variable were computed adjusting for sequencing depth as a covariate; the resulting depth‐adjusted associations remained nonsignificant (all adjusted *p* > 0.05), indicating that the null subgroup associations reported below are not artifacts of depth heterogeneity.

From the TCGA‐STAD cohort, we identified 33 GC patients with both primary tumor samples and matched adjacent normal tissue samples available for analysis. To quantify tumor–normal differences in cscRNA abundance for each patient, we calculated fold change (FC) as the ratio of tumor cscRNA count to normal tissue cscRNA count. Log_2_ transformation was applied to obtain log_2_ fold change (log_2_FC) values. Positive log_2_FC values indicate higher cscRNA abundance in tumor tissue, while negative values indicate higher abundance in normal tissue. Patients were categorized into “increaser” (FC > 1, or log_2_FC > 0) and “decreaser” (FC < 1, or log_2_FC < 0) groups based on the direction of tumor–normal changes.

To assess overall tumor–normal differences while controlling for interpatient variability, we employed the Wilcoxon signed‐rank test, a nonparametric paired test appropriate for nonnormally distributed data. To examine whether tumor–normal cscRNA dynamics were associated with clinicopathological features, we stratified log_2_FC values by tumor stage and histological grade and applied the Kruskal–Wallis *H* test for multigroup comparisons.

### 2.4. Machine Learning–Based Feature Selection for Stage‐Associated Genomic Regions

We analyzed 310 primary gastric tumor samples with complete AJCC stage information (Stages I–IV) from the TCGA‐STAD cohort. The resulting expression matrix (103 regions × 310 samples) served as input for machine learning analysis, with AJCC stage as the prediction target.

To identify reproducible candidate prognostic genomic regions while avoiding overfitting, we implemented a sequential three‐layer feature selection strategy combining statistical testing, machine learning algorithms, and stability validation.

Layer 1 (univariate statistical screening): We applied two complementary methods to identify regions with significant stage associations. First, the Kruskal–Wallis *H* test was performed for each region to assess expression differences across AJCC stages, with false discovery rate (FDR) correction to control multiple testing. Second, mutual information (MI) analysis quantified the nonlinear dependency between each region′s expression and tumor stage. Regions passing either criterion (FDR‐adjusted *p* < 0.05 or MI above threshold) were retained for Layer 2.

Layer 2 (multivariate machine learning selection): Three complementary feature selection algorithms were applied to the Layer 1 candidates: (1) LASSO (least absolute shrinkage and selection operator) regression with L1 regularization, (2) random forest feature importance based on mean decrease in impurity, and (3) recursive feature elimination (RFE) with logistic regression. For each method, regions were ranked by importance scores, and the top candidates from each algorithm were combined. Intermethod concordance was assessed using Spearman correlation of importance rankings.

Layer 3 (bootstrap stability validation): To ensure robust feature selection independent of data sampling, we performed 100 bootstrap iterations. In each iteration, samples were randomly resampled with replacement, and Layer 2 selection was repeated. Regions selected in ≥ 70% of bootstrap iterations were designated as stable features and retained as final biomarkers. The selection frequency (stability score) quantifies the reproducibility of each region′s selection.

Using the final selected regions as features, we trained a random forest classifier to predict AJCC stage. Model performance was evaluated using fivefold stratified cross‐validation to ensure balanced class representation. Performance metrics including overall accuracy, per‐class precision, recall, and F1‐score were calculated. Results were visualized as a confusion matrix to illustrate classification patterns and interstage confusion.

### 2.5. Prognostic Risk Score Model

Identification of survival‐associated genomic regions: To identify genomic regions associated with overall survival, we performed univariate Cox proportional hazards regression for each of the 103 regions separately. For each region, expression levels were used as a continuous predictor variable, with overall survival time as the time variable and vital status as the event indicator. Regions with *p* < 0.10 were considered candidates. We deliberately adopted a relaxed *p* < 0.10 threshold rather than the conventional *p* < 0.05 cutoff at this univariate screening stage, on three grounds. First, individual cscRNA‐enriched regions are expected to exhibit modest individual effect sizes consistent with the polygenic nature of transcriptomic biomarkers, and overly stringent univariate filtering can prematurely discard regions whose prognostic value is only realized in multivariate combination. Second, the subsequent multivariable Cox regression and bootstrap‐based stability validation provide internal controls against overfitting that would be expected to result from an inflated candidate pool. Third, this relaxed screening and strict selection strategy are consistent with established practice in high‐dimensional prognostic signature development. To assess the impact of this thresholding choice, we performed a sensitivity analysis using the conventional *p* < 0.05 cutoff during univariate screening: Under this stricter threshold, 12 regions remained as candidates, and the resulting risk score yielded a hazard ratio (HR) of 2.42 (95% CI 1.62–3.62; log‐rank *p* = 3.18 × 10^−4^) with a mean time‐dependent area under the curve (AUC) of 0.628 (Supporting Information 4: Table S3), supporting the qualitative robustness of our prognostic signature to the screening threshold. From these candidates, we selected the Top 6 regions ranked by *p* value to construct a multiregion prognostic model. Cox regression coefficients (*β*) for each selected region were extracted for subsequent risk score calculation.

Risk score calculation and patient stratification: A composite risk score was calculated for each patient as the weighted sum of expression levels across the six selected regions, with Cox regression coefficients serving as weights: Risk score = *Σ*(*β*_*i*_ × expression_*i*_), where *β*_*i*_ is the Cox coefficient and expression_*i*_ is the log_10_‐transformed expression level for region *i*. Patients were stratified into high‐risk and low‐risk groups using the median risk score as the cutoff value. This approach ensures balanced group sizes while maximizing prognostic discrimination.

Survival analysis: Kaplan–Meier survival curves were generated to compare overall survival between high‐risk and low‐risk groups. The log‐rank test was used to assess the statistical significance of survival differences. HR and 95% confidence interval (CI) were estimated using univariate Cox regression with risk group as a binary predictor. Median survival times for each group were calculated from Kaplan–Meier estimates. Risk tables showing the number of patients at risk at different time points were included below the survival curves.

Time‐dependent receiver operating characteristic (ROC) curve analysis: To evaluate the predictive accuracy of the risk score model at specific time points, we constructed time‐dependent ROC curves for 1‐, 3‐, and 5‐year survival. For each time point *t* and each possible risk score threshold, we calculated sensitivity (true positive rate) and 1‐specificity (false positive rate) by comparing predicted risk groups against actual survival outcomes at time *t*. Patients who died at or before time *t* were considered positive cases; patients who survived beyond time *t* or were censored after time *t* were considered negative cases. The AUC was calculated for each time point using the trapezoidal rule. Mean AUC across all time points was reported as an overall measure of predictive performance.

Multivariable Cox regression analysis: To assess the independent prognostic value of the risk score, we performed multivariable Cox regression adjusting for clinical covariates. The model included the risk score as a continuous variable along with age, gender (male vs. female), tumor stage (advanced [III–IV] vs. early [I–II]), and histological grade (high [G3] vs. low [G1–G2]). To ensure comparable effect sizes, continuous variables (age and expression levels used in univariate region analysis) were Z‐score standardized (mean = 0, SD = 1) prior to Cox regression. HRs, 95% CIs, and *p* values were extracted for each variable. For univariate analysis of individual regions, separate Cox models were fit for each of the six regions using standardized expression levels. Results were visualized as a forest plot with HR point estimates and 95% CI error bars. Statistical significance was defined as *p* < 0.05.

### 2.6. Tumor Microenvironment Analysis

External validation cohort: To validate the association between cscRNA‐based risk stratification and the tumor immune microenvironment, we utilized an independent GC cohort from the GEO database. GSE122401 was selected based on the following criteria: (1) bulk RNA sequencing data from primary STAD tissues, (2) availability of molecular subtype information including microsatellite instability (MSI) status and Epstein–Barr virus (EBV) infection status, and (3) sample size meeting the minimum requirement for exploratory CIBERSORT/ESTIMATE deconvolution (acknowledging that this remains at the lower end of recommended sample sizes, as discussed in the Results section). The final cohort comprised 26 tumor samples with complete clinical annotations.

Immune cell infiltration analysis: Immune cell composition was estimated using the CIBERSORT algorithm with the LM22 signature matrix, which enables deconvolution of 22 immune cell types from bulk RNA‐seq data. For each sample, we obtained the relative proportions of immune cell populations including B cells (naive and memory), plasma cells, T cells (CD8+, CD4+ naive, CD4+ memory resting, CD4+ memory activated, follicular helper, regulatory, and gamma delta), NK cells (resting and activated), monocytes, macrophages (M0, M1, and M2), dendritic cells (resting and activated), mast cells (resting and activated), eosinophils, and neutrophils. Only samples with a CIBERSORT *p* value < 0.05 were included in downstream analysis to ensure reliable deconvolution results.

ESTIMATE algorithm analysis: The ESTIMATE algorithm was applied to infer tumor purity and the presence of infiltrating stromal/immune cells. Three scores were calculated for each sample: (1) immune score, reflecting the infiltration level of immune cells; (2) stromal score, capturing the presence of stromal cells; and (3) tumor purity, estimated based on the combined immune and stromal signatures. These scores were compared between high‐risk and low‐risk groups defined by the cscRNA‐based risk model.

Molecular subtype stratification analysis: To explore the influence of molecular subtypes on cscRNA–immune relationships, samples were stratified by MSI status (MSI‐H vs. MSS/MSI‐L) and EBV infection status (EBV‐positive vs. EBV‐negative) based on the clinical annotations provided in GSE122401. Immune cell proportions and ESTIMATE scores were compared across these molecular subtypes using appropriate statistical tests.

Statistical methods for TME analysis: Comparisons of immune cell proportions and ESTIMATE scores between high‐risk and low‐risk groups were performed using the Mann–Whitney *U* test for nonnormally distributed continuous variables. Spearman correlation analysis was used to assess associations between cscRNA risk scores and immune cell proportions. For visualization, heatmaps were generated using hierarchical clustering with Euclidean distance and complete linkage. Log_2_FC was calculated as log_2_(mean_high_risk/mean_low_risk) to quantify differential enrichment of immune cells between risk groups. Statistical significance was defined as *p* < 0.05.

### 2.7. Somatic Mutation and Copy Number Variation (CNV) Analysis

To characterize the somatic mutation landscape associated with cscRNA‐based risk stratification, we obtained whole‐exome sequencing (WES)–derived somatic mutation data for the TCGA‐STAD cohort from the Genomic Data Commons (GDC) Data Portal. WES‐derived mutation data were available for 430 TCGA‐STAD patients, which is larger than the 307‐patient RNA‐seq cohort used for risk score construction because WES coverage in TCGA‐STAD extends to a broader patient set than RNA‐seq. The full 430‐patient mutation landscape is displayed in the oncoplot of Supporting Information 1: Figure S1A for descriptive context, whereas the high‐risk versus low‐risk group comparisons (tumor mutation burden [TMB], gene‐level mutation enrichment, and CNV) were restricted to the patients who had both WES and RNA‐seq‐based risk score assignments. Mutation calls were generated using the MuTect2 pipeline aligned to the GRCh38 reference genome. Nonsilent mutations (missense, nonsense, frame‐shift insertions/deletions, splice site, and in‐frame insertions/deletions) were retained for downstream analysis. TMB was calculated as the total number of nonsilent somatic mutations divided by the estimated exome capture size (~30 Mb). Patients were classified as TMB‐high or TMB‐low using the median TMB as the cutoff. To assess the association between gene‐level mutation frequency and risk group, we applied two‐tailed Fisher′s exact tests for the 15 most frequently mutated genes, comparing mutation rates between high‐risk and low‐risk groups. Odds ratios (ORs) with 95% CIs were calculated, and the Haldane correction (addition of 0.5 to zero cells) was applied when necessary. CNV data were obtained from the GDC Data Portal using the DNAcopy segmentation workflow. Segment mean values (log_2_ ratio) overlapping the six cscRNA‐enriched genomic regions were extracted and compared between risk groups using the Wilcoxon rank‐sum test. Mutation landscape visualization was generated using the R package maftools (oncoplot); mutation enrichment was displayed as lollipop charts using ggplot2; CNV distributions were visualized as violin plots using ggplot2 with the patchwork layout package.

### 2.8. Software and Statistical Analysis

All analyses were performed using Python with scipy.stats and statsmodels packages. Survival analyses utilized the lifelines package, including CoxPHFitter for Cox regression models and KaplanMeierFitter for Kaplan–Meier estimation. Log‐rank tests were implemented using the lifelines.statistics module. ROC curve analysis utilized the scikit‐learn metrics module. Somatic mutation analysis utilized the R packages maftools, ggplot2, and ComplexHeatmap. All visualizations were created using matplotlib, seaborn, and R/ggplot2, following standard scientific publication formatting guidelines.

### 2.9. Cell Culture

The GC cell line HGC‐27 was purchased from the Cell Bank of the Chinese Academy of Sciences (Shanghai, China), and the cells were cultured in RPMI‐1640 medium (Gibco, United States) containing 10% fetal bovine serum (Gibco, United States) with 1% penicillin–streptomycin double antibody (Invitrogen, United States). Culture conditions were 37°C, 5% CO_2_. Cells were periodically tested for mycoplasma using the MycoAlert assay kit (Lonza, Switzerland) to ensure contamination‐free. Cells in the logarithmic growth phase were used for all experiments.

### 2.10. RNA Extraction and cDNA Synthesis

Total RNA from HGC‐27 cells was extracted using TRIzol reagent (Invitrogen, United States) according to the manufacturer′s instructions. RNA concentration and purity were determined by a NanoDrop 2000 spectrophotometer (Thermo Fisher Scientific, United States), and integrity was verified by 1.5% agarose gel electrophoresis. Two micrograms of total RNA was reverse‐transcribed to cDNA using the PrimeScript RT Reverse Transcription Kit (TaKaRa, Japan) under the following reaction conditions: 15 min at 37°C and 5 s at 85°C. The reverse transcription products were stored at −20°C for spare parts.

### 2.11. RT‐PCR Validation

Six representative cscRNAs (cscR‐553, cscR‐976, cscR‐642, cscR‐863, cscR‐819, and cscR‐496) were selected for experimental validation. Specific primers were designed for the predicted cross‐strand junction sites of each cscRNA, and the primers spanned the junctions to ensure specific amplification. The PCR reaction was performed using PrimeSTAR HS DNA polymerase (TaKaRa, Japan) in a reaction mixture consisting of 2 *μ*L of cDNA template and 0.5 *μ*M each of the forward and reverse primers. The PCR program was as follows: predenaturation at 98°C for 2 min, 98°C for 10 s, 55°C–60°C (adjusted according to the *T*_m_ value of the primers) for 15 s, and 72°C for 30 s/kb for 35 cycles and final extension at 72°C for 5 min. The PCR products were analyzed by 2% agarose gel electrophoresis and stained with GelRed (Biotium, United States).

### 2.12. Genomic DNA (gDNA) Control Experiments

To distinguish whether cscRNA was a posttranscriptional product or a result of genomic rearrangement, we simultaneously extracted gDNA from HGC‐27 cells as a control. The gDNA was extracted using the DNeasy Blood & Tissue Kit (QIAGEN, Germany) according to the standard procedure. gDNA was used as the template for amplification using the same primers and PCR conditions as those for cDNA, and the source of cscRNA was determined by comparing the amplification results of cDNA and gDNA.

### 2.13. Sanger Sequencing Verification

The successfully amplified cscR‐819 RT‐PCR products were purified by gel recovery using QIAquick Gel Extraction Kit (QIAGEN, Germany) according to the instructions. The purified products were sent to Sangon Bioengineering (Shanghai) Co. for bidirectional Sanger sequencing. Sequencing primers used the same pair of primers amplified by PCR. Two independent biological replicates were sequenced to ensure the reliability of the results. Sequencing profiles were analyzed using Chromas software (Technelysium, Australia), focusing on the predicted trans‐strand junction site sequences to verify the accuracy of bioinformatic predictions.

### 2.14. siRNA Transfection

HGC‐27 cells were added into six‐well plates, and transfection was performed when the cell density was about 50%. Before transfection, the culture medium was changed to one without double antibody. Add siRNA to 200 *μ*L jetPRIME (Polyplus‐transfection SA, Illkirch, France) buffer and mix well (the final concentration of siRNA is 50 nM). Four microliters of jetPRIME reagent was added to the transfection solution and incubated for 15 min at room temperature. Add the transfection solution into the culture medium and mix well. siRNA sequence is shown in Supporting Information 2: Table S1, synthesized by Beijing Xianghong Biotechnology Co.

### 2.15. Cell Viability Assay

HGC‐27 was added into a 96‐well plate at 3000/100 *μ*L. Twelve hours later, transfection reagent was added, and 10 *μ*L of CCK‐8 reagent was added into each well after incubation for 0, 24, 48, and 72 h. After incubation for 2 h, the absorbance of the samples at 450 nm was measured using an enzyme marker.

### 2.16. Clone Formation Assay

HGC‐27 cells were treated with the transfection reagent for 24 h and then inoculated in six‐well plates at 1000 cells/well. The medium was changed every 4 days. After 12 days of incubation, the medium was discarded, washed twice with PBS, fixed with 4% paraformaldehyde for 10 min, stained with 0.1% crystal violet for 15 min, washed twice with PBS, and photographed.

### 2.17. Scratch Experiment

HGC‐27 cells were treated with a transfection reagent for 24 h and inoculated into a six‐well plate at an inoculation density of about 90%. After being cultured for 12 h to allow the cells to adhere, a “+” scratch was made using the tip of a 200 *μ*L pipette. The cells were then washed with PBS to remove floating cells and photographed at 0 h. Six‐well plates were added with 2% FBS complete medium and continued to incubate for 24 and 36 h before taking photos. The scratched area was statistically analyzed by ImageJ software.

### 2.18. Transwell Migration Assay

After adding HGC‐27 cells to the transfection reagent for 24 h, 5 × 10^4^ cells were taken and added to 200 *μ*L of serum‐free medium and inoculated into the upper chamber. At the same time, 650 *μ*L of medium with 10% FBS was added to the lower chamber. After incubation for 48 h, the chambers were washed once using PBS, the cells were fixed with 4% paraformaldehyde for 10 min, stained with 0.1% crystal violet for 15 min, and photographed, and the number of migrated cells was statistically analyzed using ImageJ software.

### 2.19. Detection of Apoptosis by Flow Cytometry

Cells and their culture medium were collected 72 h after transfection by centrifugation using EDTA‐free trypsin digestion, centrifuged at 1000 r/min for 5 min, the supernatant was discarded, and the cells were gently resuspended with 1 mL PBS, centrifuged at 1000 r/min for 5 min, and the supernatant was discarded. Apoptosis was detected using Annexin V‐FITC/PI Apoptosis Detection Kit (7Sea Biotech, Shanghai).

### 2.20. Subcellular Hierarchical Cell Separation

To study the subcellular localization of cscR‐819, we used a hierarchical separation technique to classify HGC‐27 cells into four components: cytoplasm (CY), nuclear periphery (NP), nuclear matrix (NM), and chromatin (CH). The specific steps were as follows: HGC‐27 cells in the logarithmic growth phase were collected, and the CY and nucleus were first separated using the NE‐PER Nuclear and Cytoplasmic Extraction Kit (Thermo Fisher Scientific, United States). Subsequently, nuclei were further separated into NP, NM, and CH fractions using the Nuclear Graded Separation Kit (Active Motif, United States). Each step was performed according to the manufacturer′s instructions, and all operations were performed at 4°C to maintain protein stability.

### 2.21. Western Blot Validation of Isolation Purity

The purity of the isolation of each subcellular fraction was verified using western blot. Protein samples were quantified by the BCA method (Pierce, United States), and 30 *μ*g of protein was upsampled to 10% SDS‐PAGE gel electrophoresis for separation and transferred to a PVDF membrane (Millipore, United States). Incubation was performed using the following primary antibodies: anti‐GAPDH (1:5000, Cell Signaling Technology, United States), anti‐U1‐70K (1:2000, Abcam, United Kingdom), anti‐Histone H3 (1:3000, Cell Signaling Technology, United States), and anti‐Lamin A/C (1:2000, Cell Signaling Technology, United States). Chemiluminescence detection was performed using the ECL luminescence kit (Bio‐Rad, United States) after secondary antibody incubation.

### 2.22. Absolute Quantitative qPCR Analysis

To accurately determine the copy number of cscR‐819 in each subcellular fraction, we established an absolute quantitative qPCR system. A standard plasmid containing the full‐length sequence of cscR‐819 was first constructed, and a standard curve (10^1^–10^7^ copies/*μ*L) was prepared by gradient dilution. Detection was performed on an ABI 7500 real‐time fluorescence quantitative PCR instrument using SYBR Green Master Mix (Applied Biosystems, United States). The reaction conditions were 95°C for 10 min, 95°C for 15 s, and 60°C for 1 min for 40 cycles. Three technical replicates were set up for each sample, and the absolute copy number of cscR‐819 in each fraction was calculated from the standard curve.

### 2.23. RNA Half‐Life (HF) and Translation Efficiency (TE) Analysis

To assess the effect of cscR‐819 on posttranscriptional regulation, we performed RNA HF and TE analyses at the whole transcriptome level. Cells were treated with Actinomycin D (5 *μ*g/mL, Sigma‐Aldrich, United States) to block new RNA synthesis, and cellular RNA extracts were collected at 0, 2, 4, 6, and 8 h time points. mRNA HF was calculated by qPCR assay. TE was determined by the ribosome profiling (RPF) technique, calculated as TE = RPF (ribosome − protected fragment)/mRNA abundance. Differential analysis was performed using the R package DESeq2, and cumulative distribution curves were plotted.

### 2.24. De Novo Peptide Chain Synthesis Assay

Protein synthesis levels were assayed using the puromycin doping assay. HGC‐27 cells transfected with si‐ctrl and si‐cscR‐819 were treated with 10 *μ*g/mL puromycin (InvivoGen, United States) for 30 min to adulterate nascent peptide chains. Cells were collected and fixed with 4% paraformaldehyde, permeabilized with 0.1% Triton X‐100, and stained using PE coupling with antipuromycin antibody 12D10 (1:500, Millipore, United States). PE fluorescence signal intensity was detected by a BD FACSCalibur flow cytometer (BD Biosciences, United States), and the data were analyzed using FlowJo software. Three biological replicates were set up for each group.

### 2.25. Polyribosome Analysis

Polyribosome analysis was performed by sucrose density gradient centrifugation. 1 × 10^7^ cells were collected and lysed with a lysis buffer containing 100 *μ*g/mL actinidione (Sigma‐Aldrich, United States). The supernatant was layered onto a 10%–50% sucrose gradient and centrifuged at 39,000 rpm for 2.5 h using an SW 41 Ti rotor (Beckman Coulter, United States). Fractions were collected using a gradient fractionator (ISCO, United States) while monitoring the absorbance at 254 nm. Free ribosomal subunits (40S and 60S), monoribosomes (80S), and polyribosomal regions were identified based on the absorption peaks.

### 2.26. Polyribosomal RNA Sequencing Analysis

RNA from the polyribosomal region was collected and extracted using TRIzol LS reagent (Invitrogen, United States). RNA quality was assayed by an Agilent 2100 Bioanalyzer (Agilent Technologies, United States), and samples with an RIN value of > 8 were used for library construction. Sequencing libraries were constructed using NEBNext Ultra II Directional RNA Library Prep Kit (New England Biolabs, United States), and 150 bp bipartite sequencing was performed on the Illumina NovaSeq 6000 platform. Sequencing data were aligned to the human reference genome (GRCh38) using STAR, quantified using featureCounts, and analyzed for differential expression using DESeq2. Differential gene heatmapping was performed using the R package pheatmap, focusing on functional gene categories associated with cell proliferation, apoptosis, and cancer.

## 3. Result

To systematically characterize cscRNA detection patterns in GC, we analyzed 372 samples from the TCGA‐STAD cohort, including 331 primary tumor samples and 41 solid tissue normal samples. Analysis revealed a fundamental distinction between tumor and normal tissues in their relationship with sequencing depth (Figure 1A). In primary tumor tissues, cscRNA number demonstrated a significant positive correlation with raw sequencing reads (*R* ^2^ = 0.202, *p* < 0.001), whereas adjacent normal tissues exhibited no significant correlation (*R* ^2^ = 0.023, *p* = 0.339), indicating tissue‐specific regulation of cscRNA abundance. To determine whether demographic or clinicopathological features influenced cscRNA detection within tumors, we stratified samples by gender, age, tumor stage, histological grade, and lymph node metastasis status. Remarkably, cscRNA distribution patterns remained consistent across all subgroups examined. Gender comparison showed no significant difference between male (*n* = 213, median = 53) and female (*n* = 118, median = 67; Mann–Whitney *U* test, *p* = 0.1068) patients (Figure 1B). Similarly, four age groups (< 50, 50–64, 65–74, and ≥ 75 years) displayed comparable cscRNA levels (median range: 43–94; Kruskal–Wallis test, *H* = 7.21, *p* = 0.0654) (Figure 1C). Clinicopathological analyses further confirmed this universal pattern: AJCC tumor stage (Stages I–III; *H* = 3.28, *p* = 0.1939) (Figure 1D), histological grade (G1–G3; *H* = 3.35, *p* = 0.1873) (Figure 1E), and lymph node metastasis status (N0 vs. N+; *U* = 9911, *p* = 0.3528) (Figure 1F) all showed no significant association with cscRNA abundance. Because tumor cscRNA counts correlated with sequencing depth (Figure 1A), we additionally verified that none of these subgroup associations changed when sequencing depth was included as a covariate: Raw sequencing read counts did not differ significantly across subgroups (all *p* > 0.10), and partial Spearman correlations between cscRNA counts and each clinical variable adjusting for sequencing depth remained nonsignificant (all adjusted *p* > 0.05; see Methods), confirming that the null subgroup findings are not artifacts of depth heterogeneity. Collectively, these findings demonstrate that while cscRNA detection is distinctly enhanced in tumor versus normal gastric tissues, it represents a fundamental and universal molecular feature of GC that is independent of disease progression, tumor aggressiveness, demographic characteristics, and metastatic capacity.

**Figure 2:**
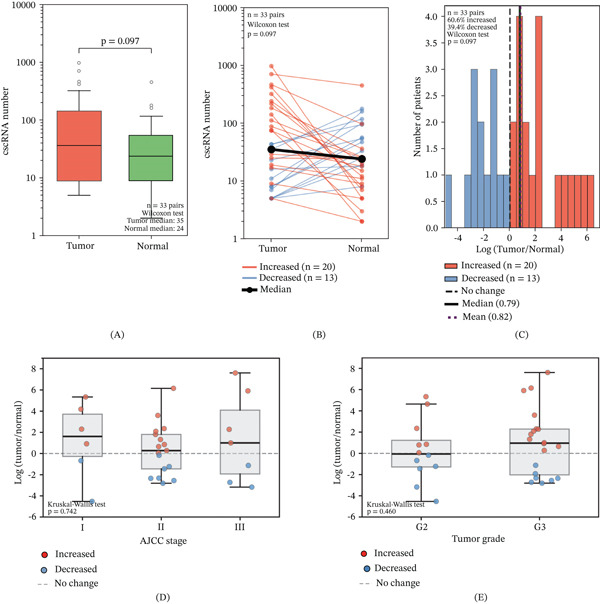
Patient‐specific heterogeneity in tumor–normal cscRNA dynamics independent of clinicopathological features. (A) Paired comparison of cscRNA abundance between matched tumor and adjacent normal tissues from gastric cancer patients. Boxplots show the distribution of cscRNA numbers in primary tumor (red) and paired solid tissue normal samples (green), with individual data points overlaid as dots. Box boundaries represent 25th and 75th percentiles; the center line represents the median; whiskers extend to 1.5× the interquartile range. The *y*‐axis is displayed on a log_10_ scale. Statistical comparison was performed using the two‐tailed Wilcoxon signed‐rank test for paired samples, with *p* value indicated. (B) Individual patient trajectories connecting paired tumor (left) and normal (right) samples. Red lines, tumor‐upward direction; blue lines, tumor‐downward direction; thick black line, median trajectory. Wilcoxon signed‐rank test. (C) Distribution of log_2_ (tumor/normal) fold changes across paired samples. Red bars, log_2_FC > 0; blue bars, log_2_FC < 0; dashed vertical line, no change; solid green line, median; dotted purple line, mean. (D) Log_2_ fold changes stratified by AJCC tumor stage (I, II, and III). Dots colored by direction of change as in Figure 2C; horizontal dashed line, no change. Kruskal–Wallis test. (E) Log_2_ fold changes stratified by histological grade (G2 vs. G3). Dots colored by direction of change as in Figure 2C. Kruskal–Wallis test.

To directly assess tumor–normal cscRNA differences while controlling for interpatient variability, we performed paired analysis of 33 GC patients with matched tumor and adjacent normal tissue samples. In this paired comparison, the median cscRNA count was 35 in tumors versus 24 in matched normal tissues, with 20 of 33 patients (60.6%; 95% CI 42.1%–77.1% by Wilson method) showing higher cscRNA counts in tumor than in matched normal tissue (Figure 2A). The Wilcoxon signed‐rank test for the overall paired difference did not reach statistical significance (*p* = 0.097), and we therefore refrain from describing this result as a positive finding; we treat it instead as a nonsignificant directional difference that, given the modest sample size (*n* = 33) and the wide CIs around the per‐patient proportions, is best regarded as exploratory and hypothesis‐generating. The individual patient trajectories shown in Figure 2B illustrate substantial interpatient heterogeneity, with patients distributed in both directions relative to their matched normal tissue. For descriptive purposes only, we refer to patients with FC > 1 as showing a tumor‐upward direction (median FC 4.56) and those with FC < 1 as showing a tumor‐downward direction (median FC 0.20); these labels denote direction of change in this exploratory cohort and are not proposed as biologically defined subgroups. The distribution of log_2_FCs ranged from −4.52 to 7.61 with a median of 0.79 and an SD of 2.97 (Figure 2C), and stratification by AJCC stage and histological grade did not reveal statistically significant associations between the direction of change and clinicopathological category (stage: *H* = 0.60, *p* = 0.742, Figure 2D; grade: *H* = 0.55, *p* = 0.460, Figure 2E). Taken together, in this small paired cohort, we did not detect a statistically significant overall enrichment of cscRNAs in tumors, and the directional heterogeneity observed at the individual patient level should be confirmed in larger paired cohorts before drawing biological conclusions about distinct cscRNA dynamic subgroups.

To enhance analytical robustness, we employed a region‐based aggregation strategy, defining 5000‐bp genomic bins and quantifying cumulative cscRNA activity across 331 TCGA‐STAD primary tumors. This identified 103 genomic regions with consistent signal across samples (mean detection rate 69.6%, range 62%–76%), enabling systematic identification of recurrent cscRNA hotspots while reducing technical noise and capturing broader regulatory patterns.

**Figure 3:**
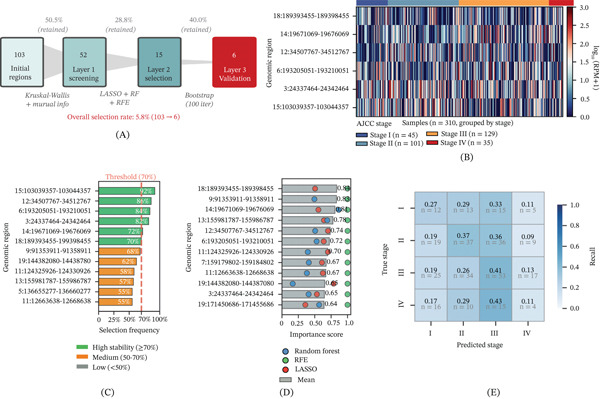
Machine learning–based identification of candidate prognostic genomic regions in gastric cancer. (A) Three‐layer feature selection pipeline for systematic reduction of cscRNA‐enriched genomic regions to candidate prognostic biomarkers. Sankey‐style flow diagram illustrating the sequential filtering process through Layer 1 (univariate screening using the Kruskal–Wallis test with FDR correction and mutual information analysis), Layer 2 (multivariate selection integrating LASSO, random forest, and recursive feature elimination algorithms), and Layer 3 (bootstrap stability validation with 100 resampling iterations applying a 70% selection frequency threshold). A color gradient from blue to red indicates progressive refinement stages from initial candidates to final high‐confidence features. Arrow widths represent the number of regions retained at each filtering step. (B) Expression heatmap of six selected genomic regions across primary gastric tumor samples stratified by AJCC pathologic stage. Column annotations indicate stage groups: Stage I (blue), Stage II (light blue), Stage III (orange), and Stage IV (red). Color intensity represents log_10_‐transformed expression levels (RPM + 1) ranging from 0 (white) to 3.00 (dark red). White vertical lines separate stage groups. Genomic regions are labeled on the *y*‐axis by chromosomal coordinates. (C) Bootstrap stability selection frequencies for top genomic regions are displayed as a horizontal bar chart. Bars show the proportion of 100 bootstrap iterations in which each region was selected by the LASSO algorithm. Bars are colored by stability level: green (≥ 70% selection frequency, high stability), orange (50%–70%, medium stability), and gray (< 50%, low stability). A red dashed vertical line marks the 70% selection threshold. The *x*‐axis represents selection frequency (0%–100%); the *y*‐axis lists genomic regions ordered by stability. (D) Multimethod feature importance scores for top genomic regions showing ensemble analysis across three machine learning algorithms. Gray bars represent mean importance scores averaged across methods; colored dots indicate individual algorithm scores overlaid on bars: LASSO (red), random forest (blue), and recursive feature elimination (green). Regions are ranked by mean importance score. The *x*‐axis represents normalized importance score (0–1.0); the *y*‐axis lists genomic regions in descending order of importance. (E) Confusion matrix evaluating stage classification performance using the six selected genomic regions via fivefold stratified cross‐validation of the random forest classifier with balanced class weights. Rows represent true AJCC stages; columns represent predicted stages. Cell colors indicate recall rates (true positive rate) ranging from 0 (white) to 1.0 (dark blue). Bold numbers show recall percentages; gray text shows sample counts. The diagonal represents correct classifications; off‐diagonal values represent misclassifications.

To identify progression‐associated regions, we applied a three‐layer machine learning pipeline (Figure 3). Layer 1 univariate screening (Kruskal–Wallis testing with FDR correction and MI analysis) identified 52 statistically significant regions (*p* < 0.05) with nonrandom stage‐specific expression patterns (Figure 3A). Layer 2 integrated LASSO, random forest, and RFE, yielding 15 high‐importance candidates with consistent intermethod rankings (*r* = 0.45–0.78; Figure 3D). Layer 3 bootstrap stability selection (100 iterations) confirmed six regions achieving ≥ 70% selection frequency (mean: 81.0%; Figure 3C).

Expression profiling across 310 samples (21 excluded for missing stage data) revealed stage‐associated patterns with notable progressive elevation in advanced tumors (Stages III–IV, expression range: 0–3.00 log_10_(RPM + 1); Figure 3B). A random forest classifier using these six regions achieved 34.2% overall accuracy via fivefold cross‐validation (Figure 3E), with per‐stage recall of 26.7% (Stage I), 36.6% (Stage II), 41.1% (Stage III), and 11.4% (Stage IV). We note that this overall accuracy (34.2%) is only modestly higher than the 25% expected under uniform random assignment to four stages and that recall for Stage IV in particular remained low. We therefore explicitly do not interpret these six regions as a clinically useful AJCC stage classifier; rather, the classification analysis indicates that these regions carry a weak but reproducible stage‐related signal that is detectable above chance, but is insufficient on its own to support stage assignment in clinical practice. The principal value of the six regions—chr18:9393455–9398455, chr14:19671069–19676069, chr12:34507767–34512767, chr6:93205051–93210051, chr3:24337464–24342464, and chr15:3039357–3044357—therefore lies not in stage classification but in their potential as candidate prognostic biomarkers, which we evaluate using survival analyses in the next section. Detailed genomic annotations for these six regions, including overlapping genes and neighboring regulatory elements, are provided in Supporting Information 3: Table S2.

**Figure 4:**
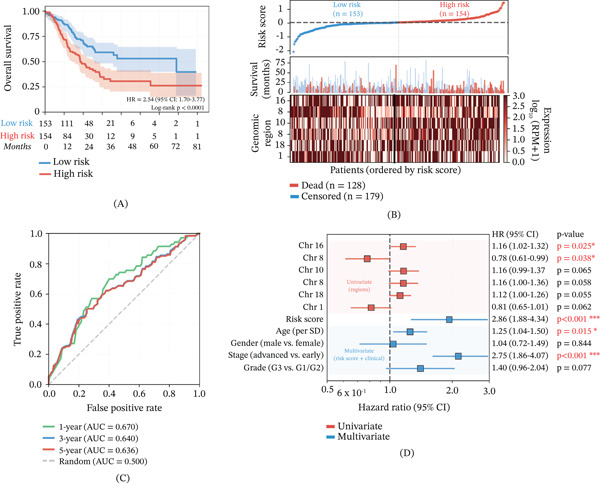
Prognostic validation of the survival‐associated genomic region risk score model. (A) Kaplan–Meier survival curves comparing overall survival between high‐risk and low‐risk patient groups. Patients were stratified based on the median risk score derived from six survival‐associated genomic regions. The red line represents the high‐risk group; the blue line represents the low‐risk group. Shaded areas indicate 95% confidence intervals. The risk table below the curve shows the number of patients at risk at different time points. Hazard ratio (HR) with 95% confidence interval and log‐rank test *p* value are shown. (B) Integrated visualization of risk score distribution, survival outcomes, and expression patterns. Top panel: Risk score distribution for all patients, ordered by increasing risk score from left to right. Blue dots represent low‐risk patients; red dots represent high‐risk patients. The horizontal dashed line indicates the median cutoff value. Middle panel: Survival status and duration for each patient are shown as vertical bars. Red bars indicate death events; blue bars indicate censored patients. Bar height represents survival time in months. Bottom panel: Expression heatmap of the six survival‐associated genomic regions across all patients. (C) Time‐dependent receiver operating characteristic (ROC) curves evaluating the predictive performance of the risk score model. Three curves represent predictive accuracy for survival at 1‐year (green), 3‐year (blue), and 5‐year (red) time points. Area under the curve (AUC) values are displayed in the legend. (D) Forest plot showing HRs with 95% confidence intervals from Cox proportional hazards regression analyses. The upper panel (red) displays univariate analysis results for each of the six individual genomic regions. The lower panel (blue) displays multivariate analysis results, including the risk score and clinical variables. Significance levels are indicated by asterisks ( ^∗^ *p* < 0.05;  ^∗∗^ *p* < 0.01;  ^∗∗∗^ *p* < 0.001).

Having identified these six candidate prognostic regions (see Methods), we next evaluated their prognostic potential using univariate Cox regression analysis. All six regions showed significant associations with overall survival, enabling the construction of a multiregion risk score model. Using the median risk score as a cutoff, patients were stratified into high‐risk (*n* = 154) and low‐risk (*n* = 153) groups (Figure 4A,B).

Kaplan–Meier analysis revealed significantly worse overall survival in high‐risk patients (median survival: 18.9 vs. 69.0 months; HR = 2.54, 95% CI: 1.70–3.77, *p* = 9.42 × 10^−5^; Figure 4A), with markedly higher event rates (51.9% vs. 31.4%, 20.5% point difference; Figure 4B). Time‐dependent ROC curves demonstrated moderate predictive accuracy at 1‐year (AUC = 0.670), 3‐year (AUC = 0.640), and 5‐year (AUC = 0.636) time points (mean AUC = 0.649; Figure 4C). Critically, multivariate Cox regression adjusting for age, gender, tumor stage, and grade confirmed the risk score as an independent prognostic factor (HR = 2.86, 95% CI: 1.88–4.34, *p* = 8.31 × 10^−7^; Figure 4D). These findings establish that the six‐region model provides robust, stage‐independent prognostic stratification for GC patients.

To investigate whether cscRNA‐based risk stratification was associated with somatic genomic alterations, we analyzed somatic mutation profiles from the TCGA‐STAD WES cohort (*n* = 430, MuTect2 pipeline; full mutation landscape provided in Supporting Information 1: Figure S1A as descriptive context). High‐risk versus low‐risk group comparisons reported below were restricted to the subset of patients with both WES data and RNA‐seq‐based risk score assignments (see Methods). Within this subset, high‐risk patients exhibited significantly elevated TMB compared with the low‐risk group (median: 5.7 vs. 3.6 mutations/Mb, Wilcoxon rank‐sum test, *p* = 1.04 × 10^−8^; Supporting Information 1: Figure S1B), and the cscRNA risk score showed a moderate positive correlation with TMB (Spearman *ρ* = 0.301, *p* = 7.22 × 10^−8^). Enrichment analysis of the 15 most frequently mutated genes (denominators are the high‐risk and low‐risk subsets defined above) revealed that several cancer‐associated genes were significantly overrepresented in the high‐risk group, including TTN (60.1% vs. 42.0%, *p* = 2.38 × 10^−4^), CSMD3 (30.3% vs. 17.0%, *p* = 1.44 × 10^−3^), PCLO (24.8% vs. 12.7%, *p* = 1.92 × 10^−3^), ACVR2A (27.1% vs. 8.5%, *p* = 5.79 × 10^−7^), and ZFHX4 (24.8% vs. 10.8%, *p* = 2.31 × 10^−4^; Fisher′s exact test; Supporting Information 1: Figure S1C). Furthermore, CNV analysis of three cscRNA‐enriched genomic regions with available segmentation data (chr12:34507767–34512767, chr14:19671069–19676069, and chr3:24337464–24342464) demonstrated differential CNV distribution patterns between risk groups (Supporting Information 1: Figure S1D). These findings indicate that cscRNA‐based risk stratification captures underlying genomic instability features, providing molecular evidence supporting the biological relevance of the prognostic model.

**Figure 5:**
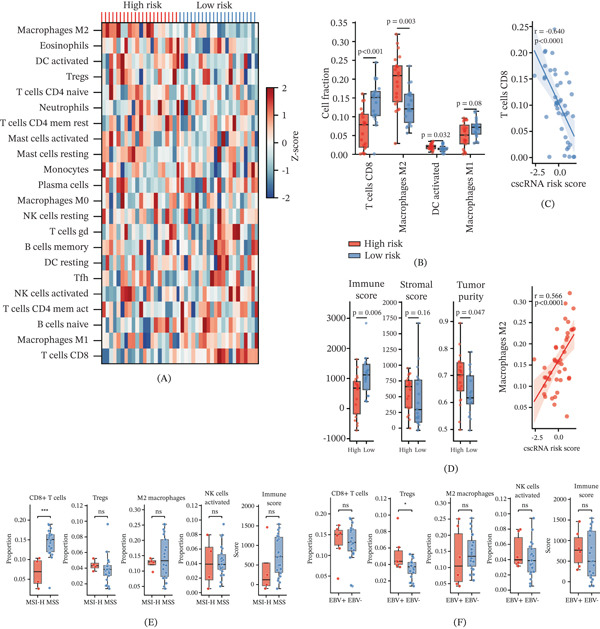
Association between cscRNA‐based risk stratification and tumor immune microenvironment in gastric cancer. (A) Heatmap of immune cell infiltration patterns in high‐risk and low‐risk groups. CIBERSORT algorithm was applied to estimate proportions of 22 immune cell types in an independent GEO validation cohort (GSE122401, *n* = 26). Rows represent immune cell types ordered by log_2_ fold change (high/low risk); columns represent individual samples grouped by risk status. (B) Boxplots comparing proportions of differentially infiltrated immune cell types between risk groups. Four immune cell populations with notable differences are displayed: CD8+ T cells, regulatory T cells (Tregs), M2 macrophages, and activated NK cells. Statistical comparison performed using two‐tailed Mann–Whitney *U* test. (C) Correlation analysis between cscRNA risk score and immune cell proportions. Scatter plots display relationships between continuous risk score (*x*‐axis) and cell proportion (*y*‐axis). Spearman correlation coefficients (*r*) and *p* values are displayed in each panel. (D) ESTIMATE algorithm analysis comparing tumor microenvironment scores between risk groups. Boxplots show the distribution of immune score, stromal score, and tumor purity in high‐risk (red) and low‐risk (blue) groups. (E) Stratified analysis of immune cell infiltration by microsatellite instability (MSI) status. Boxplots compare proportions of CD8+ T cells, Tregs, M2 macrophages, activated NK cells, and immune score between MSI‐H and MSS tumors. (F) Stratified analysis of immune cell infiltration by Epstein–Barr virus (EBV) infection status. Boxplots compare proportions between EBV‐positive and EBV‐negative tumors. In Figure 5E,F, group comparisons were performed using the two‐tailed Mann–Whitney *U* test. Asterisks indicate statistical significance:  ^∗^ *p* < 0.05,  ^∗∗^ *p* < 0.01, and  ^∗∗∗^ *p* < 0.001; ns, not significant.

To investigate the relationship between cscRNA‐based risk stratification and the tumor immune microenvironment, we performed exploratory immune infiltration analysis using CIBERSORT and ESTIMATE algorithms in an independent GEO cohort (GSE122401, *n* = 26). We acknowledge upfront that this validation cohort is modest in size; a sample of 26 tumors is at the lower end of what CIBERSORT and ESTIMATE typically require to produce stable estimates, and all results in this section should therefore be interpreted as exploratory hypothesis‐generating findings rather than as definitive validation. After applying the recommended CIBERSORT *p* < 0.05 quality filter, 26 samples passed deconvolution quality control and entered downstream analyses. Heatmap visualization of 22 immune cell types revealed distinct infiltration patterns between high‐risk and low‐risk groups, with several cell populations showing differential enrichment (Figure 5A). Comparative analysis identified four immune cell types with notable differences: CD8+ T cells showed lower infiltration in high‐risk tumors (high risk: 0.098 vs. low risk: 0.152, *p* = 0.042), while regulatory T cells (Tregs) were elevated in high‐risk cases (0.051 vs. 0.033, *p* = 0.028) (Figure 5B). Additionally, M2 macrophages and activated NK cells displayed trends toward differential distribution between risk groups.

Correlation analysis between individual immune cell proportions and cscRNA risk scores revealed significant associations (Figure 5C). CD8+ T‐cell infiltration showed a negative correlation with risk score (*r* = −0.42, *p* = 0.033), whereas Tregs exhibited a positive correlation (*r* = 0.45, *p* = 0.021). These findings suggest that high cscRNA risk is associated with an immunosuppressive microenvironment characterized by reduced cytotoxic T‐cell infiltration and increased Treg presence.

ESTIMATE algorithm analysis further supported these observations (Figure 5D). High‐risk tumors demonstrated significantly lower immune scores compared to low‐risk tumors (470.1 ± 674.7 vs. 1090.9 ± 618.1, *p* = 0.006), indicating reduced overall immune infiltration. Correspondingly, tumor purity was higher in high‐risk cases (0.69 ± 0.09 vs. 0.63 ± 0.08, *p* = 0.047), while stromal scores showed no significant difference between groups.

To explore whether molecular subtypes influenced cscRNA–immune relationships, we performed stratified analysis by MSI status and EBV infection (Figure 5E,F). Importantly, the MSI‐H subgroup and the EBV‐positive subgroup in this cohort contain very few samples (*n* = 4 and *n* = 6, respectively), and the corresponding statistical comparisons should be regarded as preliminary observations only. Because point estimates and *p* values from groups of this size are highly sensitive to individual samples, the results below are presented for descriptive purposes and are not intended to support firm biological conclusions about MSI‐ or EBV‐specific immune patterns; confirmation in substantially larger independent cohorts is essential. In MSI‐stratified analysis, MSI‐H tumors (*n* = 4) showed numerically lower CD8+ T‐cell infiltration than MSS tumors (*n* = 22) (0.067 vs. 0.142, *p* < 0.001 by Mann–Whitney *U* test); however, given that this comparison is based on only four MSI‐H samples, the apparent magnitude of the difference and the associated *p* value should be interpreted with substantial caution, and confirmation in larger MSI‐H cohorts is required. EBV stratification revealed that EBV‐positive tumors (*n* = 6) showed numerically higher Tregs than EBV‐negative cases (*n* = 20) (0.054 vs. 0.035, *p* = 0.033), again with the same caveat that *n* = 6 is insufficient to support strong biological inference. Considered together, these subgroup analyses are best regarded as exploratory observations consistent with the hypothesis that cscRNA‐based risk stratification may track aspects of tumor immune microenvironment that vary across molecular subtypes, but they do not by themselves establish that high‐risk cscRNA tumors exhibit a uniformly immunosuppressive phenotype across GC subtypes.

**Figure 6:**
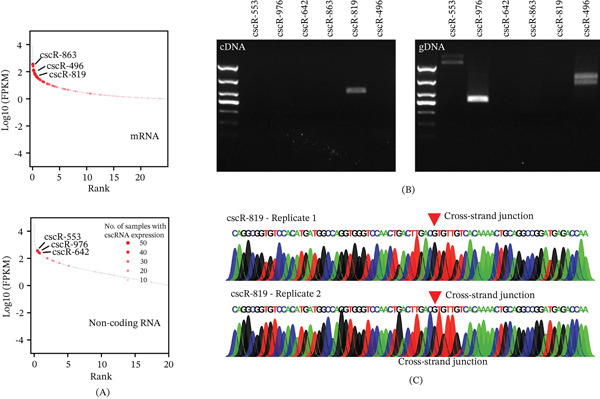
Identification and experimental validation of highly expressed cscRNAs in gastric cancer. (A) Recurrent cscRNA expression level sequencing plot. Upper panel: comparison of cscRNA and mRNA expression levels (log_10_ FPKM); lower panel: comparison of cscRNA and noncoding RNA expression levels. The size of the red dots indicates the number of samples in which the cscRNA expression was detected. (B) RT‐PCR to verify the expression of six candidate cscRNAs in the gastric cancer cell line HGC‐27. Left: PCR results with cDNA as template; right: PCR results with genomic DNA (gDNA) as template. (C) Sanger sequencing validation of the cscR‐819 cross‐strand linkage site. Sequencing results from two independent biological replicates are shown, with red triangular markers indicating the cross‐strand linkage site.

Having established the prognostic significance of the six genomic regions and characterized their association with the tumor immune microenvironment, we next sought to identify specific cscRNA molecules within these regions that may drive their functional relevance in GC. We analyzed the expression distribution of individual cscRNAs mapping to these regions and ranked them by their highest FPKM values to identify highly expressed candidates (Figure 6A).

In expression profiles compared with mRNAs (Figure 6A, upper panel), three cscRNAs showed particularly high expression levels: cscR‐863, cscR‐496, and cscR‐819, all with log_10_(FPKM) values above 2, comparable to many coding genes. Similarly, in comparison with noncoding RNAs (Figure 6A, lower panel), cscR‐553, cscR‐976, and cscR‐642 also exhibited high expression. Notably, these highly expressed cscRNAs were detected across multiple samples, with some present in > 40 samples (indicated by red dot size).

To experimentally validate these bioinformatically predicted cscRNAs, we selected six representative candidates (cscR‐553, cscR‐976, cscR‐642, cscR‐863, cscR‐819, and cscR‐496) for RT‐PCR validation in the GC cell line HGC‐27 (Figure 6B). Using cDNA as a template, only cscR‐819 amplified a clear band of expected size (left panel), while the other five showed no specific products. To confirm cscR‐819 as a posttranscriptional product rather than a gDNA rearrangement, we performed parallel PCR using gDNA as a template (right panel). cscR‐819 showed no gDNA signal, confirming its origin as a true posttranscriptional cross‐strand fusion product. In contrast, cscR‐976 and cscR‐496 detected gDNA signals, suggesting genomic rearrangement origins.

Sanger sequencing of the cscR‐819 RT‐PCR product precisely confirmed the cross‐strand linkage site (Figure 6C). Two independent biological replicates clearly showed the junction (marked by red triangles) with linkage sequence “…CTGACGTGTTGTCACAAAACTG…,” where high‐quality sequencing peaks demonstrated that “CTG” and “ACG” originated from opposite DNA strands, validating the bioinformatically predicted junction.

Given the high expression of cscR‐819 in GC and its properties as a bona fide posttranscriptional product, we further explored its functional role in the malignant behavior of GC cells. By the siRNA transfection technique, we successfully knocked down the expression of cscR‐819 in HGC‐27 cells and systematically evaluated its effects on multiple malignant phenotypes of the cells.

**Figure 7:**
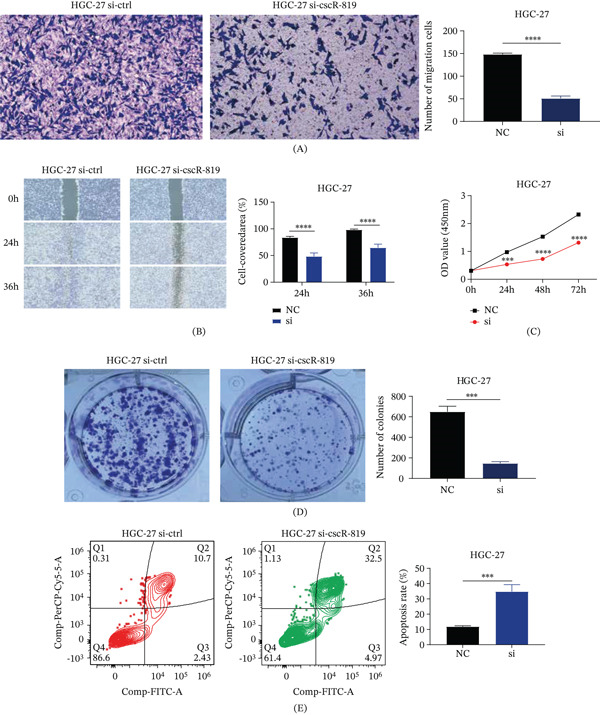
Effects of cscR‐819 knockdown on the malignant phenotype of HGC‐27 gastric cancer cells. (A) Transwell assay and (B) scratch healing assay to detect the effect of cscR‐819 knockdown on cell migration ability. (C) CCK‐8 proliferation assay and (D) clone formation assay to detect the effect of cscR‐819 knockdown on cell proliferation ability. (E) Flow cytometry to analyze the effect of cscR‐819 knockdown on apoptosis. Apoptosis was detected using Annexin V‐FITC/PI double‐staining assay. q1: necrotic cells; q2: late apoptotic cells; q3: early apoptotic cells; q4: live cells. HGC‐27 cells were transfected with si‐ctrl and si‐cscR‐819. The experiment was repeated thrice independently with similar results. Data were expressed as mean ± standard deviation, *n* = 3.  ^∗∗∗^ *p* < 0.001 and  ^∗∗∗∗^ *p* < 0.0001, using Student′s *t*‐test.

First, we evaluated the effect of cscR‐819 on cell migration ability by Transwell assay (Figure 7A). Compared with the control group (si‐ctrl), the number of cells crossing the polycarbonate membrane of the Transwell chamber was significantly reduced after knockdown of cscR‐819 (si‐cscR‐819), suggesting that cscR‐819 knockdown inhibited the migration ability of GC cells. Quantitative analysis showed that the number of cells crossing the chambers in the si‐cscR‐819 group was reduced from about 150 to about 50 in the control group (*p* < 0.001).

The results of the scratch healing assay showed that cscR‐819 knockdown significantly inhibited the cell migration ability (Figure 7B). At 0 h, the width of scratches was similar in both groups of cells. However, after 24 and 36 h, the control cells almost completely healed the scratches, while the scratches in the si‐cscR‐819 group still maintained a large gap. Quantitative analysis of the scratch healing rate further confirmed that the percentage of cellular coverage area in the si‐cscR‐819 group was approximately 50% and 65% at 24 and 36 h, respectively, which was significantly lower than that of the control group, which was 85% and 100% (*p* < 0.0001).

The CCK‐8 assay demonstrated that the knockdown of si‐cscR‐819 significantly inhibited the viability of HGC‐27 cells (Figure 7C). At 0 h, the OD values at 450 nm were comparable between the si‐ctrl and si‐cscR‐819 groups. However, the OD values for the si‐cscR‐819 group were markedly lower than those of the si‐ctrl group at 24 h (*p* < 0.001), 48 h (*p* < 0.0001), and 72 h (*p* < 0.0001). At all measured time points following 0 h, the OD values of the NC group showed a consistent and marked increasing trend, indicating active cell proliferation over time. In contrast, the siRNA‐transfected group also exhibited a gradual increase in OD values, but this increase was significantly attenuated compared to the NC group at 24, 48, and 72 h. The differences between the NC and si groups were statistically highly significant at 24 h (*p* < 0.001), 48 h (*p* < 0.0001), and 72 h (*p* < 0.0001). These results suggest that transfection with the specific siRNA significantly inhibits the proliferation of HGC‐27 cells.

Clone formation experiments showed that cscR‐819 knockdown significantly affected the cell proliferation ability (Figure 7D). The control group formed a large number of densely packed clones, while the number of clones in the si‐cscR‐819 group was significantly reduced and sparsely distributed. Quantitative analysis showed that the number of clone formations in the si‐cscR‐819 group decreased from approximately 650 in the control group to approximately 150 (*p* < 0.001).

Flow cytometry analysis revealed the regulation of apoptosis by cscR‐819 (Figure 7E). After knockdown of cscR‐819, the apoptosis rate increased from about 13% (Q2 + Q3 : 10.7*%* + 2.43*%*) to about 37% (Q2 + Q3 : 32.5*%* + 4.97*%*) in the control group, suggesting that cscR‐819 deletion promoted apoptosis. Statistical analysis of the apoptosis rate showed that knockdown of cscR‐819 increased the apoptosis rate from approximately 11% to approximately 34% (*p* < 0.001). Taken together, the results of these functional experiments suggest that cscR‐819 plays an important role in maintaining the malignant phenotype of GC cells. Its knockdown significantly inhibited the invasion, migration, and proliferation abilities of the cells, while promoting apoptosis.

**Figure 8:**
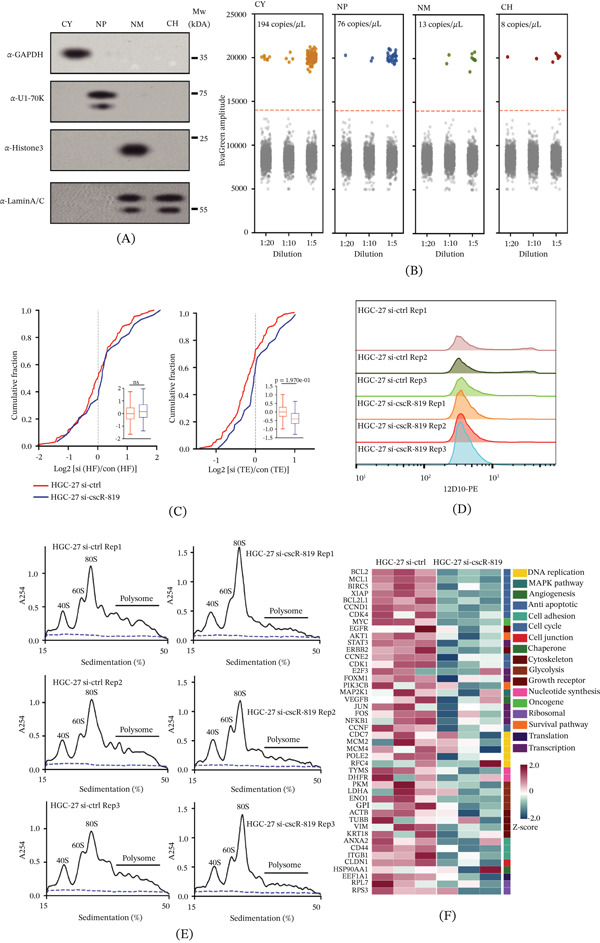
Subcellular localization of cscR‐819 and its effect on mRNA half‐life (HF) and translation efficiency (TE). (A) Western blot to verify the purity of subcellular fractions isolated from HGC‐27 cells. CY: cytoplasm; NP: nuclear periphery; NM: nuclear matrix; CH: chromatin. Marker proteins used GAPDH (cytoplasmic marker), U1‐70K (nuclear marker), Histone 3 (chromatin marker), and Lamin A/C (nuclear membrane marker). (B) Absolute quantitative qPCR analysis of the distribution of cscR‐819 in each subcellular fraction. Copy numbers at three dilutions (1:20, 1:10, and 1:5) are shown, with colored dots representing cscR‐819 and gray dots representing other transcripts. The red dashed line indicates the detection threshold. (C) Cumulative distribution curve analysis of mRNA HF and TE at the whole transcriptome level. Left panel: cumulative distribution of mRNA HF; right panel: cumulative distribution of TE. Red line: control group; blue line: cscR‐819 knockdown group. *p* value was determined by Kolmogorov–Smirnov test. (D) Flow cytometry assay for puromycin doping to assess protein synthesis levels. Shows the distribution of 12D10‐PE fluorescence intensity in three biological replicates of both the control and cscR‐819 knockdown groups. (E) Distribution of polyribosomes analyzed by sucrose density gradient centrifugation. Shows A254 absorbance curves of three biological replicates of both the control and cscR‐819 knockdown groups. The 40S and 60S ribosomal subunits and 80S mono‐ and polyribosomal (polysome) regions are labeled. (F) Heatmap of RNA sequencing of polysome fractions (polysome profiling). Z‐score values of differentially expressed genes in control and cscR‐819 knockdown groups are shown. The color bar on the right indicates the functional category to which the gene belongs. Red color indicates upregulation, and blue color indicates downregulation. Each group contains three biological replicates.

To gain a deeper understanding of the mechanism of action of cscR‐819, we first analyzed its subcellular localization pattern in HGC‐27 cells. By the cell hierarchical separation technique, we divided the cells into four fractions: CY, NP, NM, and CH. Western blot analysis confirmed the purity of the fractions (Figure 8A): GAPDH was detected only in the CY, U1‐70K was present predominantly in the NP and NM, Histone 3 specifically labeled CH fractions, and Lamin A/C was distributed in both NP and CH, indicating good separation.

Absolute qPCR was used to analyze the distribution of cscR‐819 in subcellular fractions (Figure 8B), which showed that cscR‐819 was mainly enriched in the CY (194 copies/*μ*L), followed by the NP (76 copies/*μ*L), and was less abundant in the NM (13 copies/*μ*L) and CH (8 copies/*μ*L) with lower levels in the NM (13 copies/*μ*L) and CH (8 copies/*μ*L). This cytoplasmic‐dominated distribution pattern implies that cscR‐819 may be involved in posttranscriptional regulatory processes.

Given the cytoplasmic localization of cscR‐819, we further explored its effects on mRNA HF and TE at the level of the whole transcriptome. By systematic analysis of all transcripts in HGC‐27 cells, we generated cumulative distribution curves (Figure 8C). The results showed that knockdown of cscR‐819 had no significant effect on overall mRNA HF and the cumulative distribution curves of the two groups largely overlapped (left panel, ns). However, in the TE analysis, the cumulative distribution curve of the si‐cscR‐819 group displayed a leftward shift relative to the control distribution (right panel); however, the Kolmogorov–Smirnov test for this distributional shift did not reach statistical significance (*p* = 0.182). On its own, the cumulative distribution analysis is therefore suggestive but not conclusive, and we explicitly do not describe this shift as significant. Importantly, two orthogonal lines of evidence corroborate a translational role for cscR‐819 even in the absence of a significant transcriptome‐wide cumulative shift. First, the puromycin incorporation assay (Figure 8D) directly measures nascent peptide synthesis and showed reduced global protein synthesis upon cscR‐819 knockdown across three biological replicates. Second, polyribosome profiling (Figure 8E) revealed a marked redistribution from polyribosomes to monosomes in the knockdown group, consistent with reduced active translation. Considered together, these three independent assays provide convergent—though, in the case of the cumulative distribution analysis alone, not formally significant—support for a role of cscR‐819 in maintaining global TE.

To verify this finding, we used puromycin doping to directly detect the synthesis of nascent peptide chains (Figure 8D). Flow cytometry analysis showed that the puromycin fluorescence signal (12D10‐PE) was significantly lower in the cscR‐819 knockdown group compared to the three biological replicates in the control group, indicating a decrease in the overall protein synthesis level. This result is consistent with the data from whole transcriptome analysis and further confirms the important role of cscR‐819 in maintaining cellular translational activity.

To further elucidate the regulatory mechanism of cscR‐819 on translation, we assessed its effect on the ribosome–mRNA binding status by polysome analysis (Figure 8E). By sucrose density gradient centrifugation, we isolated free ribosomal subunits (40S and 60S), monoribosomes (80S), and polyribosomal (polysome) fractions.

In three independent biological replicates, the control (HGC‐27 si‐ctrl) showed a typical polysome distribution pattern with distinct 40S, 60S, and 80S peaks, as well as abundant polysome regions. In contrast, the cscR‐819 knockdown group (HGC‐27 si‐cscR‐819) exhibited a marked reduction in polyribosomal regions and a relative increase in 80S monoribosomal peaks. This shift from polyribosomes to monoribosomes suggests that knockdown of cscR‐819 results in fewer actively translated mRNAs, consistent with an overall decrease in TE.

To gain insight into the molecular mechanisms of translational regulation by cscR‐819 and the specific genes it affects, we performed RNA sequencing analysis (polysome profiling) of polyribosomal fractions. By comparing the polyribosome‐associated transcripts of control and cscR‐819 knockdown groups, we identified key genes regulated by cscR‐819 (Figure 8F).

Heatmap analysis revealed that cscR‐819 knockdown resulted in significant changes in the translation levels of several functional categories of genes. Genes that were highly enriched in polyribosomes in the control group (red) were significantly reduced after cscR‐819 knockdown (blue), indicating a decrease in their TE. These affected genes encompassed several functional categories that are closely linked to cancer progression.

The most significantly downregulated genes included DNA replication‐related genes (MCM2, MCM4, POLE2, and RFC4), cell cycle–regulated genes (CCND1, CDK4, CDK1, and CCNE2), and multiple proto‐oncogenes (MYC, EGFR, JUN, and FOS). Notably, the translation of a key component of the MAPK signaling pathway (MAP2K1) and cell proliferation–associated transcription factors (STAT3, E2F1, and FOXM1) was also significantly inhibited.

In addition, the translation levels of antiapoptotic genes (BCL2, MCL1, BIRC5, and XIAP) and angiogenesis‐related genes were significantly reduced, which is consistent with our observation that cscR‐819 knockdown promotes apoptosis and suppresses the malignant phenotype. Meanwhile, the translation of some ribosomal protein genes (RPL7 and RPS3) was also affected, suggesting that cscR‐819 may affect the overall translational capacity by regulating ribosomal biosynthesis.

Taken together, these polysome profiling data suggest that cscR‐819 selectively promotes the translation of key genes associated with cell proliferation, survival, and malignant progression. This translational regulation of specific functional genomes explains how low cscR‐819 knockdown leads to the overall suppression of the malignant phenotype of GC cells, providing evidence at the molecular level for the understanding of the mechanism of cscRNA action in cancer.

## 4. Discussion

In this study, we conducted the first systematic investigation of cscRNAs in GC, establishing their expression landscape, clinical relevance, and functional significance through integrative computational and experimental approaches. Our analysis of 372 TCGA‐STAD samples revealed four principal findings that advance understanding of this understudied RNA class in cancer biology. First, cscRNA abundance demonstrated tumor‐specific enhancement with significant correlation to sequencing depth in malignant tissues but not in normal counterparts, yet remained remarkably independent of conventional clinicopathological variables including tumor stage, histological grade, and lymph node metastasis status. This paradoxical pattern suggests that cscRNAs may reflect fundamental alterations in transcriptional regulation inherent to the transformed state rather than markers of disease progression. Second, through a genomic region–based aggregation strategy coupled with a three‐layer machine learning pipeline, we identified six cscRNA‐enriched loci that collectively formed a reproducible prognostic signature, stratifying patients into high‐risk and low‐risk groups with significantly divergent survival outcomes (median: 18.9 vs. 69.0 months; univariate HR = 2.54, *p* < 0.001). Critically, this risk score maintained independent prognostic value after adjusting for established clinical variables (multivariable HR = 2.86, *p* < 0.001), positioning cscRNA‐based signatures as promising complementary biomarkers for precision risk stratification [14, 15]. Third, in an exploratory analysis of an independent GEO cohort (GSE122401, *n* = 26), cscRNA‐based risk stratification was associated with tumor immune microenvironment features compatible with an immunosuppressive phenotype, including reduced CD8+ T‐cell infiltration and elevated Treg presence; given the modest cohort size, these observations should be regarded as exploratory and require confirmation in larger cohorts before any causal link between cscRNA dysregulation and tumor immune evasion can be inferred. Fourth, experimental validation identified cscR‐819 as a bona fide posttranscriptional cscRNA product whose depletion profoundly impaired malignant phenotypes through selective attenuation of TE for proliferation‐ and survival‐associated genes, as demonstrated by polyribosome profiling. These findings extend beyond the initial characterization of cscRNAs by Peng and colleagues [7], establishing for the first time a mechanistic link between this noncanonical RNA class and cancer‐relevant translational control programs [16, 17].

The tumor‐specific elevation of cscRNA abundance coupled with its independence from disease stage presents an intriguing biological paradox that warrants mechanistic interpretation. We propose that cscRNA enrichment reflects genome‐wide transcriptional dysregulation characteristic of the transformed state, rather than a progressive oncogenic driver. Malignant transformation is accompanied by profound epigenetic reprogramming, including widespread alterations in DNA methylation, histone modifications, and CH accessibility that can aberrantly activate cryptic promoters and bidirectional transcription units [18, 19]. Unlike stage‐associated alterations that accumulate during tumor evolution, such fundamental transcriptional deregulation may be established early in tumorigenesis and maintained throughout disease progression, analogous to early mutational events in gatekeeper genes like TP53 [20]. This interpretation is consistent with our paired tumor–normal analysis, in which 60.6% of patients (20/33) showed higher cscRNA counts in tumor than in matched normal tissue and the remainder showed the opposite direction, although the overall paired difference did not reach statistical significance (Wilcoxon signed‐rank *p* = 0.097). We emphasize that the *n* = 33 paired cohort is too small to support firm conclusions about distinct cscRNA dynamic subgroups and that any hypothesis regarding biologically defined increaser‐ and decreaser‐like subtypes requires confirmation in substantially larger paired cohorts before being treated as established biology. To overcome the technical challenges posed by low abundance and detection instability of individual cscRNAs, our genomic region–based aggregation strategy proved effective in identifying recurrent hotspots with sufficient signal‐to‐noise ratios for biomarker development. The resulting six‐region prognostic model achieved moderate predictive accuracy (mean AUC = 0.649) with robust risk stratification, though performance remained below that of established multigene signatures such as the 70‐gene MammaPrint [21] or GC‐specific classifiers [15]. Nevertheless, the independent prognostic value demonstrated in multivariable analysis (HR = 2.86, *p* < 0.001) suggests potential utility as a complementary stratification tool, particularly when integrated with clinical parameters and other molecular biomarkers in composite prognostic algorithms [22]. Future studies incorporating cscRNA signatures alongside established markers such as MSI status, PD‐L1 expression, and HER2 amplification may enhance personalized treatment decision‐making in GC management [23].

Notably, our supplementary analysis of somatic mutation profiles provided additional evidence supporting the biological validity of cscRNA‐based risk stratification. The significantly elevated TMB observed in high‐risk patients, coupled with the enrichment of mutations in genome maintenance and CH remodeling genes such as ACVR2A, ZFHX4, and CSMD3, suggests that cscRNA risk scores may reflect underlying genomic instability. This association is biologically plausible, as increased genomic instability can promote aberrant transcription—including cscRNA production—while simultaneously driving mutational accumulation. The observed correlation between higher TMB and elevated cscRNA risk also carries potential therapeutic implications, given that TMB has been identified as a predictive biomarker for immune checkpoint inhibitor response in GC. Furthermore, the differential CNV patterns detected at cscRNA‐enriched genomic loci between risk groups suggest that structural genomic alterations at these specific regions may directly influence cscRNA biogenesis. This genomic instability connection provides a mechanistic bridge between the cscRNA‐based prognostic model and the immunosuppressive microenvironment features described below, as higher mutational load and chromosomal instability have been linked to immune evasion through neoantigen‐driven immunoediting and activation of innate immune suppression pathways.

The association between cscRNA‐based risk stratification and tumor immune microenvironment characteristics provides preliminary biological insights that merit confirmation in larger cohorts. In our exploratory analysis of an independent GEO validation cohort (GSE122401, *n* = 26), high‐risk tumors showed lower immune scores and reduced CD8+ cytotoxic T‐cell infiltration alongside elevated Treg proportions, a pattern consistent with an immunosuppressive microenvironment. Because this validation cohort is modest in size and the molecular subtype subgroups (MSI‐H *n* = 4; EBV‐positive *n* = 6) are particularly small, we frame these observations as exploratory rather than as established immunobiology. With this caveat in mind, the inverse correlation between cscRNA risk score and CD8+ T‐cell infiltration is compatible with the hypothesis that cscRNA dysregulation may track an immunologically “cold” tumor microenvironment, potentially through mechanisms involving altered cytokine, chemokine, or immunomodulatory factor expression—a hypothesis that requires direct testing in larger and prospectively annotated cohorts. The subgroup‐specific patterns observed in MSI‐H and EBV‐positive tumors, similarly, are best read as hypothesis‐generating rather than as evidence that MSI status or EBV infection mechanistically modulates a cscRNA–immune axis. From a translational perspective, the immunosuppressive features of high‐risk tumors suggest potential benefit from immunotherapy combinations or strategies to enhance T‐cell infiltration, though this hypothesis requires prospective validation. Integration of cscRNA signatures with immune checkpoint inhibitor response prediction models represents a promising avenue for future investigation.

The mechanistic elucidation of cscR‐819 function revealed a previously unrecognized role for cscRNAs in posttranscriptional gene regulation, specifically through modulation of mRNA TE. Translation represents a critical control node in gene expression, with accumulating evidence demonstrating that translational dysregulation drives oncogenesis independently of transcriptional changes [16, 24]. Our polyribosome profiling data demonstrated that cscR‐819 depletion selectively impaired translation of functionally coherent gene modules essential for cancer cell fitness, including cell cycle regulators (CCND1, CDK4, CDK1, and CCNE2), DNA replication machinery (MCM2, MCM4, and POLE2), oncogenic transcription factors (MYC, EGFR, JUN, FOS, and STAT3), and antiapoptotic effectors (BCL2, MCL1, BIRC5, and XIAP). Critically, this translational attenuation occurred without corresponding changes in mRNA stability, as evidenced by unaltered mRNA HFs, indicating a direct effect on ribosome recruitment or elongation rather than transcript abundance. The cytoplasmic enrichment of cscR‐819, particularly in contrast to many nuclear‐localized long noncoding RNAs that primarily regulate transcription [25], positions it in proximity to the translation machinery. We emphasize that the experiments presented here demonstrate a global effect of cscR‐819 on TE but do not directly resolve the molecular mechanism by which this regulation is achieved. The four scenarios outlined below are therefore offered as candidate hypotheses to motivate future work; our data do not distinguish among them, and none of them is supported by the experiments reported here beyond the general observation that cscR‐819 acts in the CY to modulate translation. (i) cscR‐819 might function as a scaffold RNA that recruits translation initiation factors such as eIF4E, eIF4G, or eIF4A to enhance cap‐dependent translation of specific mRNA targets, by analogy to the lncRNA LINC00961 [26]. (ii) cscR‐819 might interact directly with 5 ^′^ or 3 ^′^ untranslated regions of target mRNAs through sequence complementarity or structural motifs, facilitating ribosome loading similar to translational enhancer elements [27]. (iii) Given that several affected genes including MYC and BCL2 harbor internal ribosome entry sites (IRESs) that enable cap‐independent translation under stress conditions [28], cscR‐819 might specifically modulate IRES‐mediated translation. (iv) Given evidence implicating N6‐methyladenosine (m6A) RNA modifications in translational control through reader proteins such as YTHDF1 [29], cscR‐819 might bear m6A modifications or interact with the m6A machinery; this remains an important open question. We stress that none of (i)–(iv) is established by the data in this manuscript, and they should be read as plausible directions rather than conclusions. Distinguishing among these hypotheses will require direct identification of cscR‐819‐interacting proteins through RNA immunoprecipitation followed by mass spectrometry (RIP‐MS) and mapping of cscR‐819‐mRNA interactions via crosslinking immunoprecipitation sequencing (CLIP‐seq). Until such mechanism‐defining experiments are completed, any specific mechanistic claim about cscR‐819 must await direct experimental support. At the molecular level, four nonmutually exclusive axes consistent with the cytoplasmic RNA‐regulator literature could underlie this selectivity: (i) recruitment of RNA‐binding proteins known to selectively activate cell cycle and antiapoptotic transcripts, including IGF2BP1–3, ELAVL1/HuR, and YBX1; (ii) modulation of the eIF4F initiation complex (eIF4E, eIF4G, and eIF4A) on mRNAs harboring 5 ^′^ TOP or PRTE motifs, a class enriched for cell cycle genes; (iii) facilitation of cap‐independent translation through the validated IRES elements present in CCND1, CDK4, BCL2, and XIAP; and (iv) shared GC‐rich, structured 5 ^′^ UTR features that could provide a common docking platform for a cscR‐819‐containing ribonucleoprotein complex. RIP‐MS, enhanced crosslinking immunoprecipitation (eCLIP), and RPF on cscR‐819 knockdown cells will be required to discriminate among these scenarios. Regardless of the precise molecular mechanism, our findings establish cscRNAs as functional regulators rather than mere transcriptional noise, expanding the repertoire of cancer‐associated noncoding RNAs beyond well‐characterized classes such as microRNAs, long noncoding RNAs, and circular RNAs [30, 31].

Despite these advances, several limitations warrant acknowledgment and inform future research directions. First, the experimental validation rate was modest, with only one of six computationally predicted high‐expression cscRNAs (cscR‐819) confirmed as a bona fide posttranscriptional product in HGC‐27 cells. This low validation rate likely reflects multiple factors, including bioinformatic false positives inherent to fusion transcript detection algorithms [32], expression levels below RT‐PCR detection thresholds, and cell line–specific expression patterns that may not recapitulate primary tumor biology. This underscores the critical need for improved computational pipelines with enhanced specificity and orthogonal validation strategies across multiple GC cell lines and patient‐derived organoid models [33]. Second, while our polyribosome profiling revealed the translational consequences of cscR‐819 depletion, the direct molecular mechanism remains incompletely defined. Future studies employing RNA immunoprecipitation sequencing (RIP‐seq) and eCLIP are essential to identify cscR‐819‐interacting proteins and target mRNAs, respectively [34]. Third, functional characterization was limited to in vitro cell line models; validation in patient‐derived xenograft (PDX) models and genetically engineered mouse models would strengthen causal inferences and assess in vivo therapeutic potential [35]. Fourth, our study utilized retrospective TCGA data; prospective validation cohorts with standardized RNA collection protocols and long‐term follow‐up are needed to confirm the clinical utility of cscRNA‐based prognostic signatures prior to translation into clinical decision support tools. Fifth, all functional validation experiments in this study were performed in a single GC cell line, HGC‐27, which represents an undifferentiated gastric carcinoma phenotype. GC is well known to be molecularly heterogeneous, encompassing at least four distinct molecular subtypes (EBV‐positive, MSI, genomically stable, and chromosomal instability) defined by the TCGA. The functional findings reported here regarding the effects of cscR‐819 knockdown on proliferation, migration, apoptosis, and TE should therefore be regarded as observations specific to the HGC‐27 context, and we do not claim that they generalize uniformly across all GC subtypes. Validation in additional cell lines representing different molecular subtypes (e.g., AGS for chromosomal instability, NCI‐N87 for HER2‐amplified tumors, and KATO‐III for diffuse‐type tumors) and in patient‐derived organoids will be essential before broader conclusions can be drawn about the role of cscR‐819 in GC biology generally. Sixth, the immune microenvironment analyses were performed in an external cohort (GSE122401) of only 26 tumors, with the MSI‐H and EBV‐positive molecular subgroups containing only four and six samples, respectively; the immune findings, particularly the subgroup‐specific patterns, should therefore be interpreted as exploratory and require confirmation in substantially larger cohorts. Seventh, although cscRNA counts in tumor tissues correlated with sequencing depth, the clinical subgroup comparisons reported in Figure 1 remained nonsignificant after explicit checks for depth distribution across subgroups and after depth‐adjusted partial correlation analyses (see Methods); we nevertheless acknowledge that residual sensitivity to library preparation and depth normalization choices cannot be fully excluded from a single‐cohort analysis. More broadly, the contrast between tumors and matched normals—cscRNA counts correlate positively with sequencing depth in tumors but show no detectable depth dependence in normals—is most parsimoniously interpreted as a rarefaction effect on a more diverse underlying repertoire. The cscRNA species pool in normal gastric tissue is comparatively small and is effectively saturated at typical sequencing depths, whereas the tumor cscRNA repertoire is substantially larger and remains undersampled, so additional reads continue to recover novel species. This interpretation is consistent with the broader transcriptional diversification of cancer (genomic instability, aberrant splicing, and tumor cellular heterogeneity) and reframes the tumor depth correlation as a sampling consequence of biological diversity rather than as a technical confounder of the subgroup comparisons (already verified via depth‐adjusted partial correlations; see Methods). Eighth, the HGC‐27 cell line used in this study was obtained from the Cell Bank of the Chinese Academy of Sciences without independent in‐house reauthentication by short tandem repeat (STR) profiling at the time of experimentation; routine STR reauthentication of cell line identity is recommended for follow‐up work to further safeguard against cell line misidentification or cross‐contamination. Despite these limitations, our findings open promising translational avenues. The six‐region cscRNA signature could be developed into a quantitative PCR‐based assay for risk stratification in clinical laboratories, potentially integrated with existing prognostic tools such as the AJCC staging system and molecular classifiers. Moreover, the functional requirement for cscR‐819 in maintaining malignant phenotypes nominates it as a potential therapeutic target. Antisense oligonucleotide (ASO) technologies, which have achieved clinical success in treating spinal muscular atrophy and Duchenne muscular dystrophy [36], represent a viable strategy for cscRNA‐targeted therapy pending confirmation of tumor‐specific essentiality and favorable safety profiles. We further note that CIBERSORT proportions reflect immune cell abundance rather than effector function. The cytolytic activity (CYT) score (geometric mean of GZMA and PRF1; [37]), together with broader cytotoxicity (GZMB, IFNG, GNLY, and NKG7) and exhaustion (PDCD1, LAG3, HAVCR2, and TIGIT) panels, provides a complementary functional readout. We hypothesize that the reduced CD8+ infiltration in high‐risk cscRNA tumors may co‐occur with reduced effector function and elevated exhaustion, in which case the high‐risk subgroup would correspond to an immunologically “cold” phenotype defined jointly by quantity and function; direct testing in larger immune‐profiling cohorts is a priority for follow‐up work. Whether the cscRNA enrichment and immunosuppressive associations described here are gastric‐specific or reflect a broader principle of solid tumor biology remains an open question. The cscRNA detection methodology itself is not anatomically restricted [7]. The broader low CD8+/elevated Treg/low immune score phenotype is moreover shared by many immunologically “cold” epithelial tumors, providing a candidate set of cancer types in which the cscRNA–immune axis could be most informatively tested. Systematic pancancer profiling using paired transcriptomic and immune deconvolution data from TCGA cohorts (e.g., STAD, COAD, READ, LIHC, and ESCA) is an essential next step to determine whether this axis generalizes beyond GC. Looking forward, comprehensive characterization of cscRNA landscapes across diverse cancer types, investigation of cscRNA–drug response relationships, exploration of cscRNA–immune interactions, and single‐cell resolution mapping of cscRNA expression heterogeneity will further illuminate the biological significance and therapeutic potential of this emerging RNA class. In conclusion, this study establishes cscRNAs as functionally relevant contributors to GC pathogenesis through translational regulatory mechanisms and identifies exploratory associations with tumor immune microenvironment features, providing a foundation for future investigations of cscRNAs as candidate biomarkers, therapeutic targets, and potential modulators of antitumor immunity in precision oncology.

**Nomenclature**
cscRNA: cross‐strand chimeric RNA
FPKM: fragments per kilobase of transcript per million mapped reads
CY: cytoplasm
NP: nuclear periphery
NM: nuclear matrix
CH: chromatin
RPF: ribosome‐protected fragment
gDNA: genomic DNA
TMB: tumor mutation burden
CNV: copy number variation
WES: whole‐exome sequencing

## Author Contributions

All authors contributed to the final approval of the paper. S.C. and H.H. designed the study. S.C., M.C., and S.Z. conducted the experiment and made significant contributions to the manuscript writing. Y.H. and X.W. analyzed the data. X.L. offered guidance on the writing. H.H. and S.W. revised the manuscript. S.C. and M.C. contributed equally to this work as co‐first authors.

## Funding

This work was supported by research grants from the National Natural Science Foundation of China (No. 82560798), the Guiyang Second People′s Hospital (Jinyang Hospital) Youth Talent Fund Program (No. QN[2024]12), and the Key Laboratory for Chronic Disease Biomarkers of Guizhou Medical University (No. 2024fy004).

## Disclosure

All authors have read and approved the final manuscript.

## Ethics Statement

The authors have nothing to report.

## Consent

The authors have nothing to report.

## Conflicts of Interest

The authors declare no conflicts of interest.

## Supporting Information

Additional supporting information can be found online in the Supporting Information section.

## Supporting information


**Supporting Information 1** Figure S1: The somatic mutation landscape of the TCGA‐STAD cohort, including (A) the oncoplot of the full 430‐patient cohort, (B) the tumor mutation burden comparison between high‐ and low‐risk groups, (C) gene‐level mutation enrichment analysis of the most frequently mutated genes, and (D) copy number variation distributions across cscRNA‐enriched genomic regions.

**Supporting Information 2** Table S1: The siRNA sequences used for cscR‐819 knockdown.

**Supporting Information 3** Table S2: Detailed genomic annotations for the six cscRNA‐enriched prognostic regions, including overlapping genes and neighboring regulatory elements.

**Supporting Information 4** Table S3: The sensitivity analysis of the prognostic risk score under the stricter univariate screening threshold (*p* < 0.05).

## Data Availability

The raw data of this article will be available from the corresponding authors upon reasonable request.
