# Supplementary material for: Cross‐Strand Chimeric RNA Signature Predicts Prognosis and Identifies Tumor Immune Microenvironment Associations in Gastric Cancer
Source: Hum Mutat. 2026 Jun 17;2026:4428673. doi: 10.1155/humu/4428673 (PMC13276290; doi:10.1155/humu/4428673)
Supplement: Supplementary file 1 — Supporting Information 1 Figure S1: The somatic mutation landscape of the TCGA‐STAD cohort, including (A) the oncoplot of the full 430‐patient cohort, (B) the tumor mutation burden comparison between high‐ and low‐risk groups, (C) gene‐level mutation enrichment analysis of the most frequently mutated genes, and (D) copy number variation distributions across cscRNA‐enriched genomic regions. [file HUMU-2026-4428673-s001.pdf]

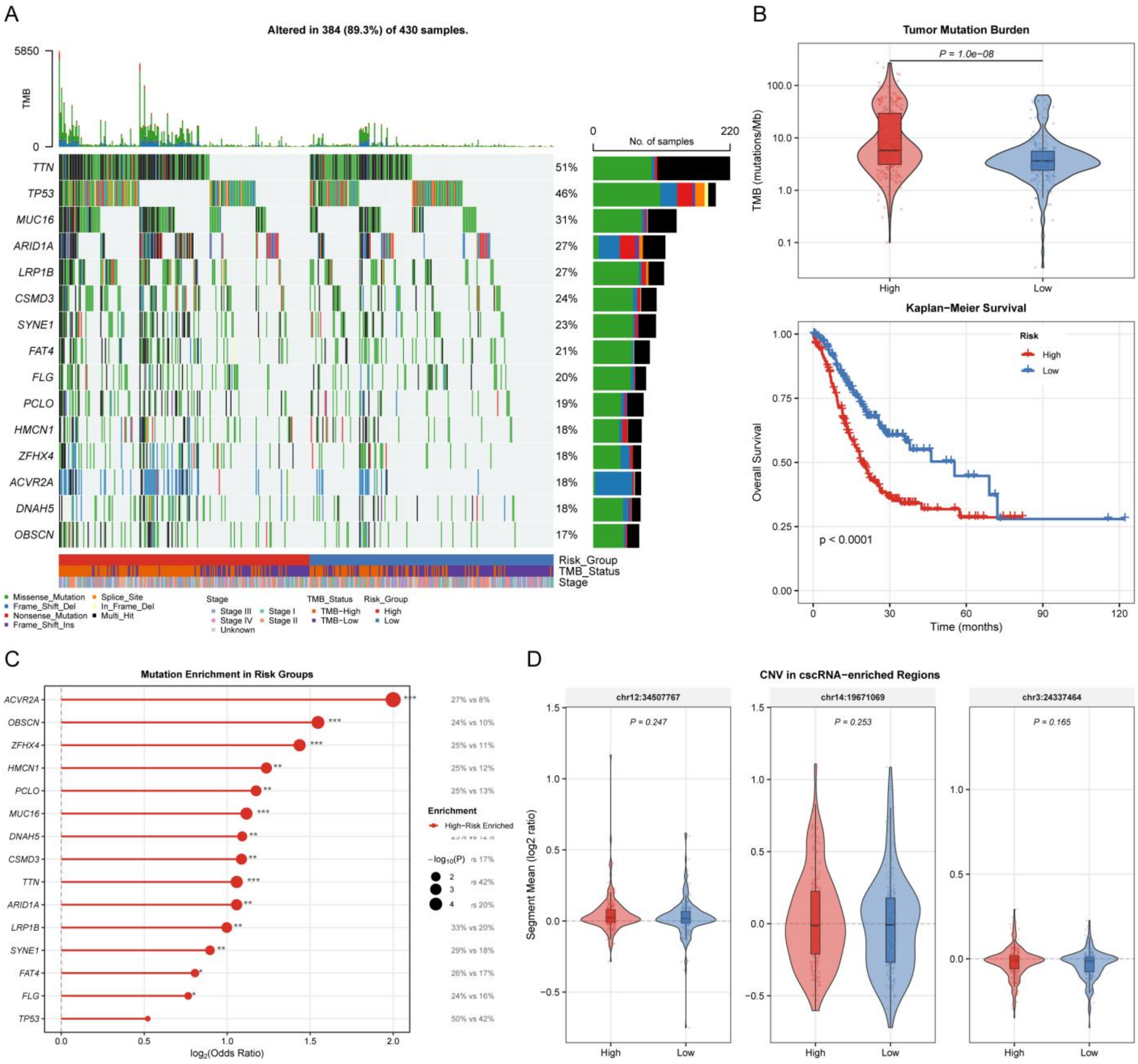

Supplementary Figure 1. Somatic mutation landscape and its association with cscRNA-based risk stratification in gastric cancer.

(A) Oncoplot displaying the somatic mutation profiles of the top 15 most frequently mutated genes across 430 TCGA-STAD patients. Each column represents one patient; each row represents one gene. Colored cells indicate mutation type: missense mutation (green), nonsense mutation (red), frame-shift insertion/deletion (brown/dark brown), splice site mutation (orange), in-frame insertion/deletion (pink/maroon), and multi-hit mutations (black). Gray cells indicate no detected mutation. Top bar plot shows the total number of non-silent mutations per patient. Right bar plot shows the mutation frequency of each gene. Clinical annotations are displayed below the main heatmap: Risk_Group (red = high-risk, blue = low-risk), TMB_Status (orange = TMB-High, purple = TMB-Low), and Stage (green = Stage I, orange = Stage II, light purple = Stage III, pink = Stage IV, gray = Unknown). Patients are sorted first by risk group, then by mutation count. Somatic mutation data were obtained from the GDC Data Portal (MuTect2 pipeline, GRCh38).

(B) Association between tumor mutation burden (TMB) and cscRNA risk stratification. Upper panel: violin plot with embedded boxplot comparing TMB distribution between high-risk and low-risk groups. Individual data points are overlaid as jitter dots. Box boundaries represent 25th

and 75th percentiles; center line represents median; whiskers extend to 1.5× interquartile range. Y-axis displayed on log10 scale. Statistical comparison performed using two-tailed Wilcoxon rank-sum test. Lower panel: Kaplan-Meier survival curves comparing overall survival between high-risk (red, n = 154) and low-risk (blue, n = 153) patient groups, based on 307 patients with complete survival data. Shaded areas indicate 95% confidence intervals. Log-rank test P-value is displayed.

(C) Lollipop chart illustrating the enrichment of somatic mutations in the top 15 most frequently mutated genes between high-risk and low-risk groups. X-axis represents $\log_2$-transformed odds ratio (OR); positive values indicate enrichment in the high-risk group (red), and negative values indicate enrichment in the low-risk group (blue). Segment length represents the magnitude of the $\log_2$(OR). Point size is proportional to $-\log_{10}$(P-value), with larger points indicating greater statistical significance. Frequency labels on the right side show mutation rates in the high-risk versus low-risk group. Significance levels are indicated by asterisks: * $P < 0.05$, ** $P < 0.01$, *** $P < 0.001$. Statistical comparison performed using two-tailed Fisher's exact test.

(D) Violin plots comparing copy number variation (CNV) profiles of three cscRNA-enriched genomic regions between high-risk and low-risk groups. The three regions—chr12:34507767–34512767, chr14:19671069–19676069, and chr3:24337464–24342464—were selected from the six previously identified stage-associated regions based on the availability of overlapping CNV segment data (DNAcopy workflow, GDC Data Portal). Y-axis represents segment mean values ($\log_2$ ratio), where positive values indicate copy number gain and negative values indicate copy number loss. Horizontal dashed line indicates the neutral state (segment mean = 0). Violin shapes represent kernel density estimation of the data distribution; embedded boxplots show median and interquartile range. Individual data points are overlaid as jitter dots. Red = high-risk group; blue = low-risk group. Statistical comparison performed using two-tailed Wilcoxon rank-sum test, with P-values displayed above each panel.
